# Priority nutrients to address malnutrition and diet-related diseases in Australia and New Zealand

**DOI:** 10.3389/fnut.2024.1370550

**Published:** 2024-03-13

**Authors:** Carlene S. Starck, Tim Cassettari, Emma Beckett, Skye Marshall, Flavia Fayet-Moore

**Affiliations:** ^1^FOODiQ Global, Sydney, NSW, Australia; ^2^School of Health Sciences, The University of New South Wales, Sydney, NSW, Australia; ^3^Research Institute for Future Health, Gold Coast, QLD, Australia; ^4^Bond University Nutrition and Dietetics Research Group, Faculty of Health Sciences and Medicine, Bond University, Robina, QLD, Australia; ^5^The Centre for Health Services Research, The University of Queensland, St Lucia, QLD, Australia; ^6^School of Environmental and Life Sciences, The University of Newcastle, Callaghan, NSW, Australia

**Keywords:** priority nutrients, malnutrition, diet-related disease, inadequate nutrient intake, increased nutrient requirements

## Abstract

**Background:**

The double burden of malnutrition and diet-related disease has been attributed to diets high in ultra-processed and discretionary foods, with increased sugars, saturated fats, and sodium, and insufficient dietary fibre. There is a limited understanding of the role of other macronutrients and micronutrients.

**Objective:**

Determine the highest priority nutrients to address both malnutrition and diet-related disease in Australia and New Zealand, for each demographic group and the total population.

**Methods:**

A novel four-step methodological approach was undertaken to identify: 1. Demographic (age-sex) groups; 2. Health priorities; 3. Potential nutrients based on inadequacy, increased requirements, and health priority association; and 4. Priority nutrients. Nutrient intake data was obtained from the most recent Australian and New Zealand nutrition surveys. Health priorities were based on national statistical data and expert consultation. High-level scientific literature (systematic reviews) was scoped for associations with health priorities and the suitability of recommended intakes. A quantitative scoring matrix was developed and used to determine the highest priority nutrients, with scoring over three domains: extent of inadequacy; consensus for increased requirements; and degree of association with health priorities.

**Results:**

Nutritional inadequacies were common, with 22 of 31 essential nutrients consumed below recommended levels. Nine priority nutrients were identified across the demographic groups, with each demographic group characterised by a specific subset of these. Six nutrients were highest priority within the total population: vitamin D, calcium, omega-3 fatty acids, magnesium, folate, dietary fibre.

**Conclusion:**

The extent of nutritional inadequacies in Australia and New Zealand is high, both within each demographic group and the entire population, relative to both recommended intakes and key health outcomes. The methodology can be applied to other countries and globally. Findings make a significant contribution to understanding the nutrients to prioritise in future-proofing the health of the Australian and New Zealand populations. Guidelines and policies can target priority nutrients to address the malnutrition and diet-related disease double burden.

## Introduction

Malnutrition, defined by the World Health Organization as deficiencies, excesses, or imbalances in a person’s intake of energy and/or nutrients, contributes to a double burden within the developed world, where an increasing proportion of the population are either not meeting recommended nutrient intakes, suffering from diet-related chronic diseases, or both (1, 2). The relationship between adequate nutrition and the maintenance of health is well-established, with each nutrient playing important and integrated roles in metabolism and biochemistry, contributing to recommended levels of dietary intake to maintain health (3, 4). While most often attributed to excess caloric intake, diet-related disease pathogenesis also involves nutrient inadequacies, where intake and/or status is suboptimal (1, 2). This includes (but is not limited to) cardiovascular disease (CVD), type 2 diabetes (T2DM), obesity, some cancers (1, 2), and infectious disease, including COVID-19 (5, 6). Although some diet-related diseases show elevated prevalence across the population, demographic groups have specific nutrient requirements and diet-related health priorities, indicating that the relative importance of each nutrient may vary depending on age and sex, and for women, pregnancy and menopausal status. For example, vitamin D deficiency or insufficiency has been suggested to increase the global burden of disease overall (7), but has also been associated with osteoporosis in menopausal women (8), an increased risk for gestational diabetes in pregnant women (9), and increased susceptibility to respiratory infection in the older men and women (10). Folic acid supplementation is recommended pre- and during pregnancy due to the well-established increased risk of sub-optimal folate for foetal neural tube defects (11, 12).

The increase in diet-related disease prevalence in the developed world, including Australia and New Zealand, has been primarily attributed to the widespread over-consumption of foods that are both energy rich and nutrient poor (2, 13, 14), partly due to a large percentage of the diet made up of ultra-processed and/or discretionary foods and beverages in these countries. These foods are predominantly high in saturated fat, sodium, and added sugars, nutrients labelled to be of concern and recommended to limit across dietary guidelines globally (13). The diets also fail to meet food group recommendations and are consequently inadequate in meeting several macro- and micronutrient recommendations (13, 15); in Australia and/or New Zealand, common nutrient inadequacies include calcium, vitamin D, iron, selenium, zinc, folate, thiamin, and vitamin B12 (16–18). Other factors impacting suboptimal dietary choices include (but are not limited to) affordability and sustainability, with refined ingredients often cheaper than their whole-food counterparts (19), and an increasing movement towards plant-based diets, containing, for example, processed meat and dairy alternatives (20). There is evidence that increasing intake of key micronutrients has a measurable impact on public health, for example, via mandatory fortification of bread with folic acid to prevent neural-tube defects (12) and the universal iodisation of salt and bread to prevent iodine deficiency-related hypothyroidism and goitre (21, 22). Thus, there is a need to identify the nutrients that offer the greatest potential to address both malnutrition and diet-related disease in Australia and New Zealand.

Nutritional adequacy plays a key role in supporting health priorities for each demographic group. Reference intake values for each essential nutrient are provided by government recommendations, such as the US Dietary Reference Intakes (DRIs) (23), and the Nutrient Reference Values (NRVs) for Australian and New Zealand (3). The NRVs were developed to determine the average nutrient values needed by individuals and populations, with the majority last updated in 2006; the most recent update was 2017 (for sodium). While the NRVs provide intake recommendations for essential nutrients across the lifespan to maintain health, varying according to life stage, age, and sex, scientific evidence suggests that they may be outdated, with some nutrient requirements greater than prescribed (24, 25). Further, the NRVs include suggested dietary targets (SDTs) for some nutrients, where higher nutrient intake recommendations are provided to help support and prevent chronic disease, showcasing the importance of nutrients beyond overt malnutrition. For some of these nutrients, doses shown to reduce the risk of chronic disease (26) are at doses greater than those in the NRVs. Thus, there is a need to better understand the evidence-base related to nutrients of the highest priority for frank deficiency prevention, reduction of diet-related disease, and health promotion. This information plays an important role in informing food policy, dietary guidelines, food development and fortification programs to close nutrient gaps, prevent deficiency, improve the quality of the food supply, and better support public health across the lifespan.

The aim of this research was to develop and implement methodology for determination of the highest priority nutrients for each demographic group (based on age and sex, in alignment with the NRVs) in Australia and New Zealand. These nutrients can simultaneously support both nutrient adequacy and efforts to reduce diet-related disease. Further, the methodology can be applied globally to uncover and compare nutrient inadequacies and priority nutrients between countries.

## Methods

For each demographic group, nutrient intake information from the most recent Australian [2011/12 (18)] and New Zealand [2008/09 (27), 2002 (28)] nutrition surveys were obtained, followed by the identification of health priorities, based on government-managed statistical and consultation with an expert (nutritional and dietary management) panel of PhD-qualified health care professionals. A detailed scope of high-level scientific evidence regarding the relationship of specific nutrients to health priorities was performed (systematic literature reviews (SLRs) of randomised controlled trials (RCTs) or prospective cohort studies), including any evidence that current recommended intakes may not be sufficient to support health (SLRs, RCTs, and nutrient balance studies). A quantitative scoring matrix was developed and used to determine the nutrients with the highest priority for each demographic group. For each nutrient and each demographic group, the scoring was based on: 1. the level of dietary inadequacy [percentage of the population with intake below the Estimated Average Requirement (EAR) or Adequate Intake (AI)]; 2. the presence of consistent evidence for increased requirements (compared to current EAR/Recommended Dietary Intake (RDI)/AI values); and 3. the degree of association with identified health priorities.

**Figure 1:**
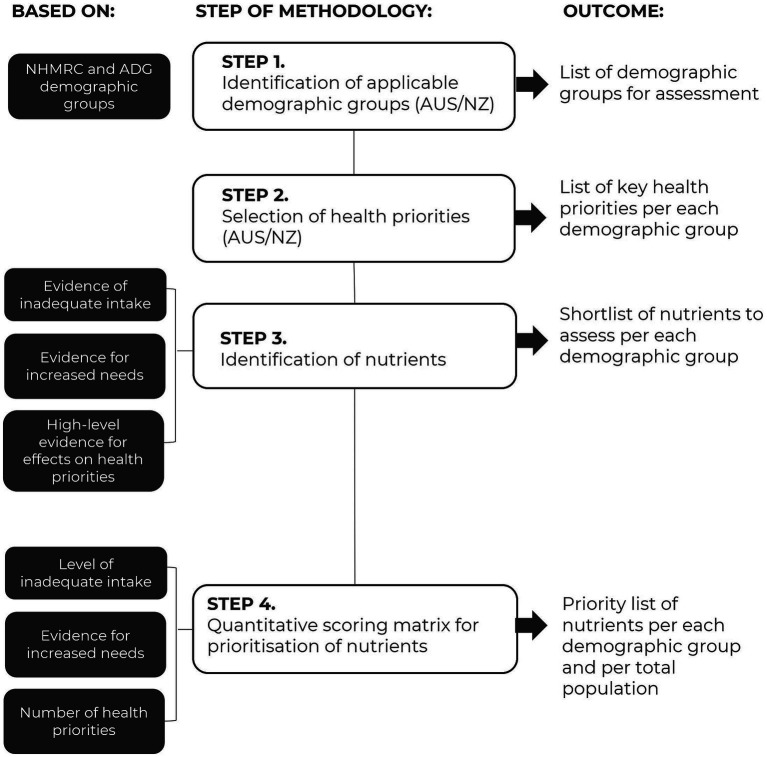
Flowchart of methodology applied to identify priority nutrients per each demographic and at the population level.

An overview of the methodology used is provided as a flowchart in Figure 1. The methodology included 4 steps to identify demographic groups for assessment, followed by the key health priorities, a shortlist of nutrients, and a final list of priority nutrients, per each demographic group. An assessment of priority nutrients for the total population was also performed. These steps are described in detail below.

### Step 1: Demographic selection

Demographic groups were created based on both consistency and clinical relevance in health priorities and nutrient requirements, taking age and sex into account. The age groups used by the National Health and Medical Research Council’s (NHMRC) Nutrient Reference Values (NRVs; targets for both Australia and New Zealand) (3) and dietary modelling to inform the Australian Guide to Healthy Eating (29) were assessed for suitability and refined as follows. The NHMRC age groups were collapsed if the following conditions were met: no clinically relevant difference in the nutrient requirements between consecutive age groups; no significantly different eating patterns known between consecutive age groups; and, health priorities were consistent between consecutive age groups. Age groups were split if health priorities were substantially different for a subsection of the age group, e.g., women of peri or post-menopausal age. Males and females were combined for age groups when the following criteria were met: no clinically relevant difference in the nutrient requirements; and, health priorities were consistent between the sexes. Infants and toddlers under 4 years of age were excluded due to a focus on children and adults.

### Step 2: Selection of health priorities

Health priorities were determined by a targeted search of government-managed statistical information followed by consultation with a panel of healthcare professionals. Health priorities were categorised as the leading causes of death (30), non-fatal physical morbidity (31–34), and mental or cognitive ill-health (34, 35) that are known to be modifiable by diet. The health priorities in each category and for each demographic group were first informed by nationally representative Australian data collected by the Australian Bureau of Statistics (ABS), the Australian Department of Health, and the Australian Institute of Health and Welfare (AIHW). Health priorities identified as a subtype of a larger disease were reclassified as the umbrella disease most relevant to dietary intervention; for example, lung cancer as cancer, coronary heart disease as cardiovascular disease, Alzheimer’s disease as dementia, back pain as chronic pain. Australian data were used to determine the health priorities for both Australia and New Zealand, due to Australia being considered broadly representative of the health and dietary intake patterns of New Zealand (36–38), based on a larger population size, having equivalent NRVs to New Zealand (3), and due to the leading causes of death or morbidity being comparable between Australia and New Zealand (39, 40).

The top 50% of health priorities for each demographic group were finalised via workshop consultation with a panel of five PhD-qualified health professionals with expertise in the nutritional and dietary management of the demographic groups. Health priorities with the highest prevalence of mortality and/or morbidity and highest association with diet were prioritised. In addition to confirming the data-derived leading causes of mortality and morbidity, the health professionals were able to advise on any additional health priorities not identified by the scientific literature.

### Step 3: Identification of the nutrients

Nutrients considered for inclusion in the assessment (Table 1) were defined as those recognised by the NRVs (3). Energy was excluded due to being dependent on more than one nutrient, including those already listed. Sodium, saturated fat, and sugars were excluded due to being known nutrients of excess and concern in the population. Water was excluded due to the impact of additional beverages, including those containing nutrients, on hydration.

**Table 1:** Nutrients considered for inclusion.

Nutrient type	Nutrients considered
Macronutrients	Protein, dietary fibre, n-6 (linoleic) fatty acids, n-3 (ALA, DHA, DPA, EPA) fatty acids.
Vitamins	Vitamins A, B_1_, B_2_, B_3_, B_6_, B_12_, C, D, E and K; folate, pantothenic acid, biotin, choline.
Minerals	Calcium, chromium, copper, fluoride, iodine, iron, magnesium, manganese, molybdenum, phosphorus, potassium, selenium, zinc.

ALA, alpha-linolenic acid; DHA, docosahexanoic acid; DPA, docosapentanoic acid; EPA, eicosapentanoic acid; n-3, omega 3; n-6, omega 6.

For each nutrient, three types of evidence were used, for: inadequate intake (percentage of the population with intake less than the EAR or AI); increased needs (evidence for a requirement greater than the current EAR, RDI, AI), and an established association to modify one of the identified health priorities. For completeness, any evidence for excess intake [above the Upper Level of Intake (UL)], decreased needs, and an adverse association with health priorities was also captured.

#### Evidence of inadequate intake

Dietary intake data were extracted from the 2011–2012 Australian National Nutrition and Physical Activity Survey (18), the 2008/09 New Zealand Adult Nutrition Survey (27), and the 2002 New Zealand National Children’s Nutrition Survey (28). These data represent the most recent national survey data available for Australia and New Zealand, obtained from 24-h dietary recall data collected via in-person interviews with trained interviewers. Interviews with children were conducted in the presence of a caregiver, either in the child’s home or at school. As age ranges used in New Zealand intake data were not equivalent to NRV age groupings, data were integrated with the most closely matched NRV age grouping. Where survey data were not available (for example, for pregnant and lactating women), a targeted literature search via the PubMed (Medline) database for peer-reviewed original research of nationally representative Australian or New Zealand samples was conducted and used as a proxy.

The level of intake of each nutrient, relative to its NRV (EAR, AI, or UL if applicable) for each demographic group was recorded, as well as the percentage of the population with intake lower than the NRV, if applicable. Intakes higher than the UL were recorded, in the instance that any nutrient was identified as being of concern (beyond the established nutrients of concern: sodium, saturated and trans fats, and added sugars). Nutrients were classified as having inadequate intake for a demographic group if 20% or more of that group had an intake below the EAR. Where a nutrient was defined by an AI, inadequate intake was defined as 50% or more of the population having an intake below the AI, in recognition that the AI cannot be used to determine inadequacy, yet represents the best indicator of requirement for some nutrients (41). Nutrients were classified as having excess intake for a demographic group if 20% or more of that group had an intake above the UL. Deficiency data according to nutritional status (serum or urinary data) were used in place of the EAR/AI where intake information was not available. Where the intake or deficiency data differed between Australia and New Zealand, age or sex groups, within the same demographic group, data from the segment of the demographic group with the highest proportion below the EAR or AI was used to represent that demographic group. For example, if teenagers from Australia were found to have inadequate intake for a nutrient, but New Zealanders met requirements for the same nutrient, the nutrient was classed as having inadequate intake, with the level of inadequacy for Australian teenagers taken to represent all teenagers. This process ensured that the most at-risk group was identified and would be accounted for. Where percentage inadequacy or percentile data were not available, the mean population intake was compared to the NRV (EAR or AI) to determine inadequacy, with a mean or median intake below the NRV taken to represent ≥50% with intakes below the NRV. Nutrients of concern were defined as those with a 20% or more prevalence of intake above an upper level of intake.

#### Increased requirements

Evidence of increased nutrient requirements was defined in three ways: 1. the nutrient is known to have limited absorption, utilisation, or increased losses in the demographic group; 2. the nutrient has an established SDT [recommended intake to prevent a high burden of chronic disease (3)]; 3. there is a consensus in the scientific literature that the NRV (EAR, RDI, or AI) is too low for a demographic group, or that the demographic group may benefit from intakes above the specified NRV. Evidence was obtained in the first instance via a search of the information provided by the NRV report. However, given many of the NRVs have not been updated since 2006, a targeted literature search of peer reviewed original research was undertaken, limited to SLRs, RCTs, and nutrient balance studies. Any evidence for decreased requirements or that a demographic group would benefit from intakes lower than the specified NRV was captured when applicable.

#### Established association with health priority

To collate evidence showing an association of a nutrient with one of the identified health priorities, a targeted literature search of the PubMed database was undertaken, complemented by a grey literature search for guidelines, on 18 November 2022. The search strategies used to scope the health priorities are listed in Supplementary Table S1. Only SLRs of RCTs or prospective cohort studies were eligible for inclusion to ensure that the highest level of available evidence was captured, based on the NHMRC framework (42). Where the evidence-base was extensive, the most recently published studies were prioritised. For each SLR selected, the evidence was applied to the appropriate demographic group(s) according to the age ranges covered by the included research. Dosage information was extracted and reported where available, including dose–response relationships and any dose associated with adverse effects.

### Step 4: Determination of highest priority nutrients

To identify priority nutrients for each demographic group, nutrients were objectively scored using a point-based scoring matrix at each of three levels, and scores added to determine an overall priority score. The scoring matrix is shown in Table 2. First, a baseline score was determined based on the extent of dietary inadequacy (percentage of sampled population having an intake below the EAR or AI) within the demographic group, from 0 (no inadequacy) to 4 (more than 80% of the sampled population having a lower than recommended intake) for the EAR, and from 0 (no inadequacy) to 2 (more than 75% of the sampled population have a lower than recommended intake) for the AI. Distinct scoring was used for the EAR vs. the AI given the limitations of using the AI for describing inadequacy (41). For nutrients where the risk of inadequacy was determined by the proportion with low nutritional status (e.g., vitamin D and iodine via serum or urinary analysis, respectively), the baseline score was determined using the same scoring system as if the nutrient had an EAR. Up to one additional point was added where the evidence strongly suggested an increased intake was optimal for health. For evidence taken from the NRVs regarding poor absorption or utilisation, or increased losses, a point was only awarded in the absence of baseline points for inadequacy, to avoid double scoring. One point was added for each health priority a nutrient showed an established beneficial association with, up to a maximum of 5 points, representing the maximum number of health priorities selected. Thus, the total score for any potential priority nutrient was 10 points. While the number of health priorities differed between demographic groups, this did not affect scoring, as nutrients were assessed for priority relative to the other nutrients within a demographic group only.

**Table 2:** Quantitative scoring matrix for nutrient prioritisation.

Scoring criterion	Score	Description
**Priority nutrient scoring**		
*Baseline score:* evidence of inadequate intake	0	No inadequacy
1	≥20- < 40 population less than EAR*OR* ≥ 50- < 75% population less than AI
2	≥40- < 60% population less than EAR*OR* ≥ 75–100% population less than AI
3	≥60- < 80% population less than EAR
4	≥80% population less than EAR
*Additional points:* evidence of increased needs^a^	0	No or equivocal evidence
	1	Consistent evidence or consensus for increased needs
*Additional points:* evidence of established association with health priority	0	No associated health priorities
	1	One associated health priority
	2	Two associated health priorities
	3	Three associated health priorities
	4	Four associated health priorities
	5	Five associated health priorities
**Nutrient of concern scoring**		
*Baseline score:* evidence of excess intake	0	No excess
	1	≥20- < 40% population greater than UL
	2	≥40- < 60% population greater than UL
	3	≥60- < 80% population greater than UL
	4	≥80% population greater than UL
*Additional points:* evidence of decreased needs^a^	0	No or equivocal evidence
	1	Consistent evidence or consensus for decreased needs
*Additional points:* evidence of established adverse association with health priority	0	No associated health priorities
	1	One associated health priority
	2	Two associated health priorities
	3	Three associated health priorities
	4	Four or more associated health priorities
	5	Five associated health priorities
**Total score**		**Priority nutrient score minus nutrient of concern score**

a Relative to the current EAR, RDI, or AI. AI, Adequate Intake; EAR, Estimated Average Requirement; UL, Upper Level of Intake.

If a nutrient was identified as having excess intake, decreased requirements, or an adverse association with a health priority, it was labelled as a nutrient of concern and scored based on the extent of dietary excess (above the UL) from 0 (no excess) to 4 (more than 80% of the population having an intake above the UL), the presence of evidence for decreased requirements (up to one additional point), and the number of health priorities where intake of a nutrient was associated was an adverse effect (up to a maximum of 5 points). These points were subtracted from the priority scoring for that nutrient to determine an overall score.

Within each demographic group, scores for each nutrient were calculated and ranked from highest to lowest. Nutrients scoring in the top quartile (25%) for each group were taken to be the highest priority nutrients for that demographic group. Where scores were tied in a way that did not readily distinguish the top 25% of nutrients, the selection of the highest priority nutrients was based on the grouping containing the number of nutrients closest to that equivalent to the top 25%. For example, in a case where there are 20 nutrients in total, five priority nutrients would be selected (25% of 20). However, if four nutrients had the highest score of four, and four nutrients had the next highest score of three, the two potential groupings would be either four or eight highest priority nutrients, respectively. Thus, according to the methodology, the top four nutrients would be selected as highest priority, since four is closer to the quartile number of five than is eight.

The process was repeated for all nutrients identified for all demographic groups (based on having inadequate intake and/or increased needs and/or an established association with health priorities) to determine the highest priority nutrients across the total Australia/New Zealand population.

## Results

### Step 1: Selection of demographic groups

Seven demographic groups were created: Children (male and female) aged 4–11 years; Teenagers (male and female) aged 12–18 years; Males aged 19–60 years; Females aged 19–45 years; Pregnant or lactating females aged 19–45 years; Peri-menopausal or menopausal women aged >45 to 60 years; and, Older adults (males and females) aged >60 years. The age of ≥60 or 65 years is routinely used to define older adults in research (43). A list of the demographic groups and the rationale for their development is provided in Table 3.

**Table 3:** Demographic groups included in the current research and their health priorities.

Demographic group	Rationale for group development	Final selected health priorities
Children (male and female) aged 4–11 years	Children 4–8 years are grouped together in NHMRC demographic groups (3). Ages 9–11 were included due to similarities in health requirements, e.g., pre-pubertal growth.	Bone mass acquisition Cognitive development Infectious immunity
Teenagers (male and female) aged 12–18 years	Although teenage females have some changes hormonally, the majority of nutrients show similar requirements between males and females (3). Health priorities were specified based on sex where applicable.	Anxiety and depression Cognitive development Growth and development focusing on bone and muscle Maturation focusing puberty/sexual organ development
Males aged 19–60 years	Male demographic groups specified by the NHMRC from ages 19–60 years have predominantly similar nutrient needs (3).	Anxiety and depression Cancer CVD Metabolic disorders
Females aged 19–45 years	To cover females of reproductive age; female demographic groups specified by the NHMRC from ages 19–45 years have predominantly similar nutrient needs (3).	Anxiety and depression Cancer CVD Fertility and pre-pregnancy nutrition Metabolic disorders
Pregnant or lactating females aged 19–45 years	Pregnancy and lactation life stages have specific nutrient requirements (3).	Healthy foetal/infant growth and development. Healthy maternal weight gain. Maternal mental health.
Peri-menopausal or menopausal females aged >45–60 years	To cover the menopausal transition, including perimenopause and menopause (44). These life stages are accompanied by unique biological changes.	Anxiety and depression Bone health Cancer CVD Metabolic disorders
Older adults (males and females) aged >60 years	Grouped from >60 years rather than >70 years [as defined by NHMRC (3)] due to the majority of women being post-menopausal at this age (44), and for consistency between males and females. In addition, the age of 60–65 years and older is usually used to define older adults in research (43).	Cognitive function / decline CVD Physical independence (i.e., muscle and bone maintenance, falls risk) Metabolic disorders

CVD, cardiovascular disease; NHMRC, National Health and Medical Research Council.

### Step 2: Selection of health priorities for each demographic group

The health priorities selected for each demographic group are provided in Table 3. Health priorities identified for each category (leading causes of death, non-fatal physical morbidity, mental or cognitive ill-health, or other) are listed in Supplementary Table S2. Final health priorities were primarily either specific to life stage, such as healthy maternal weight gain for pregnancy and lactation or were non-communicable diseases covering most of the adult population, such as cancer and cardiovascular disease. Some health priorities across demographic groups shared common features such as bone health but had differential focus according to life stage. For example, bone mass acquisition for children, compared to the maintenance of bone health for peri and post-menopausal woman. The number of final health priorities ranged from three to five, depending on the demographic group.

### Step 3: Identification of nutrients

Out of 31 eligible nutrients for consideration, 22 (71%) nutrients were categorized as having inadequate intake, 19 (61%) as having increased needs, and 16 (52%) as being associated with a health priority, across the Australian and New Zealand populations (Table 4; Supplementary Tables S3–S5, respectively). The demographic groups with the highest number of inadequate nutrients were males (19–60 years) and older adults (males and females, >60 years), with 16 nutrients having at least 20% or 50% of the demographic group not meeting the EAR or AI, respectively. Children had the lowest number of inadequate nutrients (*n* = 7). The demographic group with the greatest number of nutrients showing an increased need was older adults (*n* = 16) and both males and peri-menopausal/menopausal women shared the greatest number of health priority associations (*n* = 9). No nutrients of concern other than those already established as nutrients of concern in the literature (sodium, saturated fat, and added sugars), were identified as having excess intake or evidence for decreased needs. However, two adverse associations with health priorities were identified: dairy protein for males [increased risk of prostate cancer (8–10%) with an intake of at least 30 g/day and a dose–response association per 20 g/day increase (45)]; and folate for pregnant women (increased risk of gestational diabetes mellitus with the highest vs. lowest serum levels of folate or prolonged folic acid supplementation (46); however, evidence was rated as low and very low certainty, respectively) (Supplementary Table S5).

**Table 4:** Nutrients identified by type of evidence for each demographic group.

Demographic group	Identified nutrients		
	Inadequate dietary intake	Increased requirements	Established association with health priority^a^
Children (male and female) aged 4–11 years	Omega-6 fatty acids, dietary fibre, choline, potassium, calcium, fluoride, vitamin B6	Iron, protein, magnesium	Vitamin D, calcium, omega-3 fatty acids
Teenagers (male and female) aged 12–18 years	Omega-6 fatty acids, dietary fibre, vitamin A, vitamin B6, choline, calcium, fluoride, iodine, magnesium, potassium, selenium, thiamin, vitamin E, iron, phosphorus	Calcium, iron, zinc, protein, magnesium	Vitamin D, calcium, dietary fibre, monounsaturated fatty acids, omega-3 fatty acids
Males aged 19–60 years	Omega-6 fatty acids, dietary fibre, vitamin A, vitamin D, choline, calcium, fluoride, iodine, magnesium, potassium, selenium, zinc, thiamin, vitamin B6, pantothenic acid, biotin	Choline, zinc, protein, vitamin C, magnesium, Long chain omega-3 fatty acids, dietary fibre, vitamin A, vitamin E, folate, potassium	Omega-3 fatty acids, vitamin D, vitamin C, folate, zinc, magnesium, dietary fibre, selenium, omega-6 fatty acids, *protein*
Females aged 19–45 years	Dietary fibre, thiamin, vitamin B6, vitamin D, choline, calcium, fluoride, iodine, magnesium, vitamin B12, potassium, selenium	Iron, protein, vitamin C, magnesium, zinc, Long chain omega-3 fatty acids, dietary fibre, vitamin A, vitamin E, folate, potassium	Omega-3 fatty acids, vitamin D, vitamin C, folate, zinc, magnesium, dietary fibre, omega-6 fatty acids, protein
Pregnant or lactating females aged 19–45 years	Dietary fibre, vitamin B6, vitamin D, folate, choline, calcium, iodine, potassium, iron	Protein, folate, pantothenic acid, iodine, iron, selenium, zinc, magnesium	Omega-3 fatty acids, vitamin D, *folate*, iodine, calcium, zinc
Peri or post-menopausal females aged >45–60 years	Dietary fibre, thiamin, vitamin B6, vitamin D, choline, calcium, fluoride, iodine, magnesium, potassium, selenium	Calcium, protein, vitamin C, magnesium, zinc, long chain omega-3 fatty acids, dietary fibre, vitamin A, vitamin E, folate, potassium	Omega-3 fatty acids, vitamin D, vitamin C, folate, zinc, magnesium, dietary fibre, calcium, protein, omega-6 fatty acids
Older adults (males and females) aged >60 years	Omega-6 fatty acids, dietary fibre, thiamin, riboflavin, vitamin B6, vitamin D, choline, calcium, fluoride, iodine, magnesium, potassium, selenium, zinc, vitamin A, vitamin B12	Protein, vitamin B12, vitamin D, calcium, zinc, riboflavin, vitamin C, magnesium, zinc, long chain omega-3 fatty acids, dietary fibre, vitamin A, vitamin E, folate, potassium	Omega-3 fatty acids, omega-6 fatty acids, dietary fibre, protein, folate, vitamin D

a Nutrients in italics represent an adverse association identified for one or more health priorities. Beneficial associations may also have been identified for this nutrient within this demographic group.

**Figure 2:**
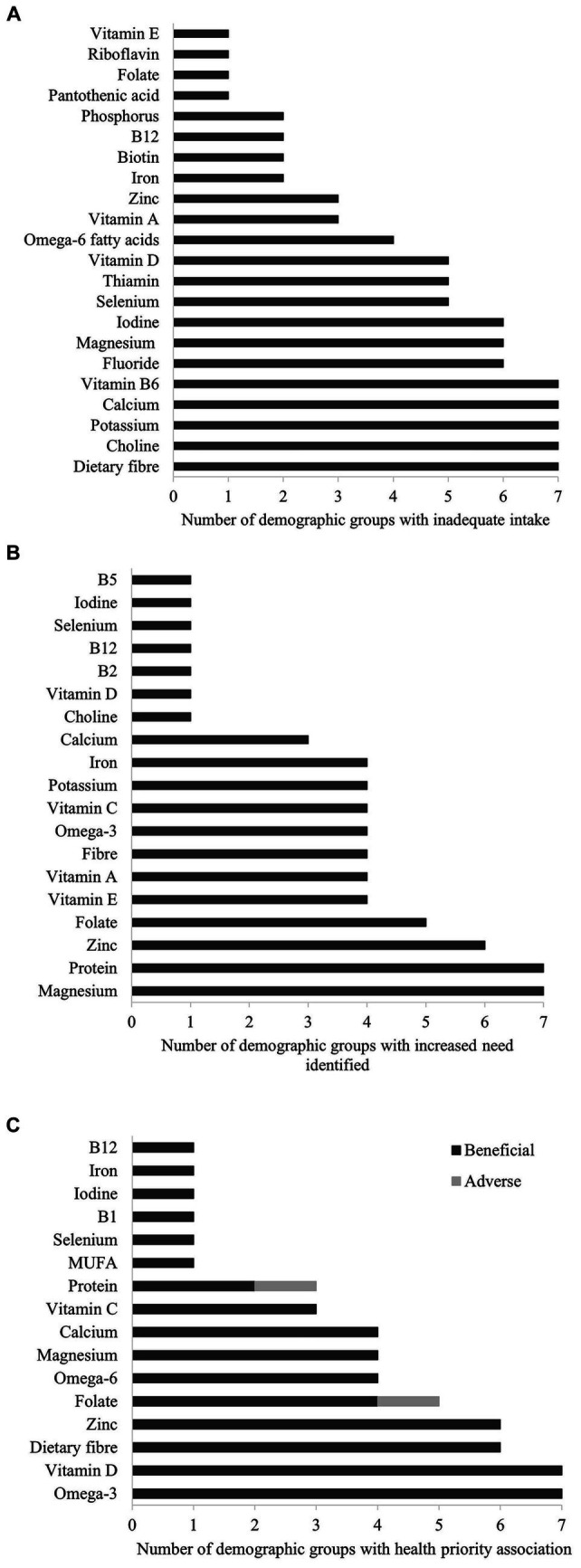
Nutrients ordered according to prevalence of inadequate intake **(A)**, increased needs **(B)**, and association with health priorities **(C)** across all demographic groups.

Figure 2 details the number of demographic groups with nutrient inadequacy, increased needs for a nutrient, and nutrient-health priority associations for the total population (all included demographic groups, from age 4 to >60 years). The highest prevalence of inadequate intake was found for dietary fibre, choline, potassium, calcium, and vitamin B6, with inadequate intake identified for all demographic groups (Figure 2A). Nutrients showing the highest prevalence for increased needs were magnesium and protein (all demographic groups), followed by zinc (*n* = 6 demographic groups) and folate (*n* = 5 demographic groups) (Figure 2B) and those with the highest number of demographic groups showing a health priority association were vitamin D and omega-3 fatty acids (all demographic groups), followed by dietary fibre and zinc (*n* = 6) (Figure 2C). While folate featured as having a health priority association in five demographic groups, this association was consistently beneficial (no adverse effects identified) in four demographic groups only.

### Step 4: Prioritisation of nutrients

**Figure 3:**
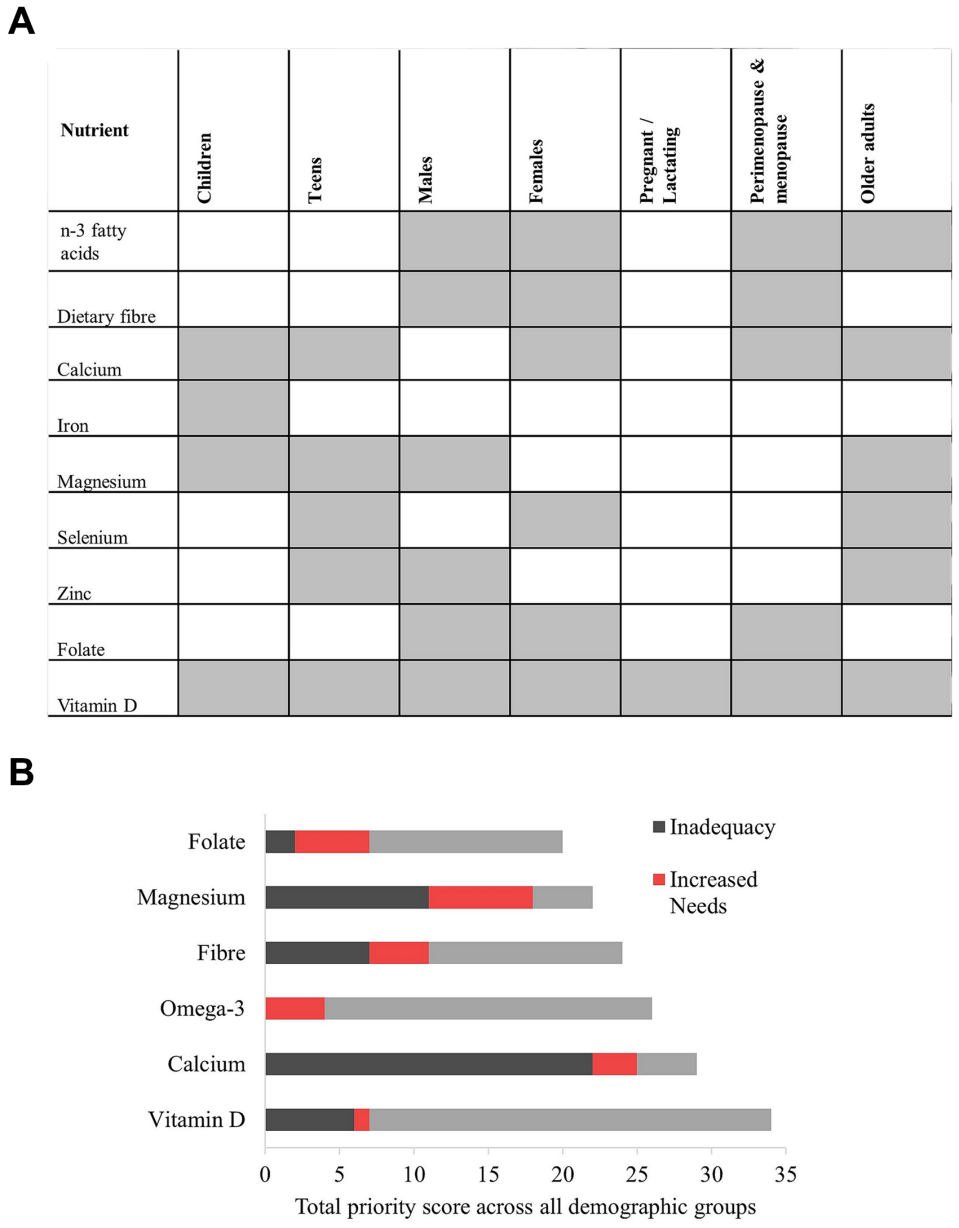
The “population-level priority nutrients”; those nutrients with the highest priority identified across the demographic groups. **(A)** Prevalence of the priority nutrients across the demographic groups; **(B)** Highest priority (top 25%) nutrients based on population-level scoring across all demographic groups. n-3, omega-3; Preg/lact., Pregnancy and lactation; Peri/meno., Perimenopause and menopause.

Between one (pregnancy and lactation) and six (males, females aged 19–45 years, and older adults) priority nutrients were identified for each demographic group. These details, including information on level of inadequacy and dose associated with health priorities is provided in Table 5. While folate was a close second to vitamin D for pregnancy and lactation, scoring was downgraded due to the adverse health association identified for this demographic group for folate. A total of nine priority nutrients featured across all demographic groups. Within this group of nine priority nutrients, those with the highest prevalence for prioritisation across the demographic groups were vitamin D (all demographic groups, *n* = 7), calcium (*n* = 5), magnesium (*n* = 4), and omega-3 fatty acids (*n* = 4) (Figure 3A).

**Table 5:** Highest priority (top 25%) nutrients identified for each demographic group, with dietary inadequacy, increased needs, and health priority association data.

Demographic group	Identified nutrients			
	Priority nutrients	Level of dietary inadequacy	Level of increased need	Associated health priorities (effective dose)
Children (male and female) 4–11 y	Calcium	EAR: 520–800 mg/day Mean intake: 676–866 mg/day 20.3% of AUS girls failed to meet the EAR (18).	N/A	Bone health and bone mass acquisition (460 mg/day increase; 300 to 1,200 mg/day) (47–49)
	Vitamin D	N/A	N/A	Infectious immunity [H v L serum status; < 300,000 IU (single dose)] (50, 51) Bone health and bone mass acquisition (132 IU/day to 14,000 IU/week) (52, 53) Cognitive development (60 to 1,000 IU/day) (54)
	Magnesium	EAR: 110–200 mg/day Mean intake: 212–252 mg/day 24.6% of AUS girls failed to meet the EAR (18).	Intake of 133 mg/d for children aged 4–8 years old necessary for growth and adequate bone mineral content (55). Magnesium requirements should be updated to account factors that can affect the need for magnesium (56).	N/A
	Iron	N/A	Increased requirement due to iron accretion during childhood growth (3).	Cognitive development (1.04 to 1.17 μg/day) (57)
Teenagers (male and female) 12–18 y	Calcium	EAR: 1050 mg/day Mean intake: 912–946 mg/day 67 to 90.3% AUS teenagers failed to meet the EAR (18). 59.1 to 87.8% NZ teenagers failed to meet the EAR (27).	N/A	Growth and development focusing on bone and muscle (460 mg/day increase; 300 to 1,200 mg/day) (47–49)
	Magnesium	EAR: 300–340 mg/day Mean intake: 252–322 mg/day Up to 71.6% of Australian teenagers did not meet the EAR (18).	Magnesium requirements should be updated to account factors that can affect the need for magnesium (56).	N/A
	Selenium	EAR: 50–60 μg/day Mean intake: 34.9–66.6 μg/day 40.4 to 78.2% of NZ teenagers did not meet the EAR (27).	N/A	N/A
	Vitamin D	N/A	N/A	Anxiety and depression (25 μg/day to 1,250 μg/week) (58) Cognitive development (60 to 1,000 IU/day) (54) Growth and development focusing on bone and muscle (132 IU/day to 14,000 IU/week) (52, 53)
	Zinc	EAR: 6–11 mg/day Mean intake: 10.6–12.8 mg/day. 27.4% of Australian males failed to meet the EAR (18).	Increased requirement for males due to higher losses in semen combined with possible decreased absorptive capacity (3, 59, 60). US requirement data may overestimate zinc absorption by 10% (60); a similar overestimation may be relevant to AUS/NZ.	Cognitive development (1.7 to 2.9 mg/day) (57)
Males 19–60 y	Dietary fibre	AI: 30 g/day Mean intake: 20.8–24 g/day 50^th^ percentile of intake was below the AI for AUS (18) and NZ (27) men.	The SDT is 38 g/day for men (3, 61).	Cancer (high vs. low intake) (62, 63) CVD (high vs. low intake; 10 g/day increase; 3–30 g/day) (64–66) Metabolic health (3–20 g/day) (66)
	Zinc	EAR: 12 mg/day Mean intake: 12.1–15 g/day 24.2 to 52% of AUS and NZ men did not meet the EAR (18, 27)	Increased requirement for males due to higher losses in semen combined with possible decreased absorptive capacity (3, 59, 60). US requirement data may overestimate zinc absorption by 13% (60); a similar overestimation may be relevant to AUS/NZ.	Anxiety and depression (7 to 25 mg/day) (67)
	Folate	N/A	The SDT for dietary folate equivalents is 300–600 μg; an additional 100–400 μg over current intakes (3).	Anxiety and depression (0.5 to 10 mg/day folic acid; 15 mg/day L-methylfolate) (68) Cancer (high vs. low intake; 20 to 30 mg/day folic acid) (69–71) CVD (0.8 mg folic acid) (72)
	Omega-3 fatty acids	N/A	The SDT for long-chain omega-3 fatty acids is 610 mg for men (3, 61).	Anxiety and depression (all doses, but ≥2000 mg/day better than <2000 mg/day) (73) Cancer (0.51 to 2.2 g/day EPA and 0.24 to 0.92 g/day DHA; high vs. low blood levels DPA or DHA) (74, 75) CVD (high vs. low blood levels DPA, DHA, EPA; 0.5 to >5 g/day; per 1 g/day increase) (72, 75–82) Metabolic health (High vs. low blood markers of omega-3 intake) (75)
	Vitamin D	AI: 5–10 μg/day Serum levels used as proxy for intake. 21.6–31.1% of AUS men showed mild to severe vitamin D deficiency (18).	N/A	Anxiety and depression (≥ 50,000 IU/week) (83) Cancer (high vs. low blood levels; 1,200 to 8,000 IU/day after cancer diagnosis) (84–88) CVD (high vs. low blood levels; 1,000 to 7142.9 IU/day) (89–92) Metabolic health (1,000 to 7142.9 IU/day; 59,000 IU periodically) (92, 93)
	Magnesium	EAR: 330 mg/day Mean intake: 366–391 mg/day 33 to 46.5% of AUS men did not meet the EAR (18).	Magnesium requirements should be updated to account factors that can affect the need for magnesium (56).	Anxiety and depression (120 to 300 mg/day elemental magnesium; 250 mg/day magnesium oxide; 320 mg to 4 g/day magnesium sulfate) (94)
Females 19–45 y	Dietary fibre	AI: 25 g/day Mean intake: 17–20 g/day 50th percentile of intake was below the AI for AUS (18) and NZ (27) women.	The SDT is 28 g (3, 61).	Cancer (high vs. low intake) (62, 63) CVD (high vs. low intake; 10 g/day increase; 3–30 g/day) (64–66) Metabolic health (3–20 g/day) (66)
	Calcium	EAR: 840 mg/day Mean intake: 724–847 mg/day The proportion of Australian and New Zealand women failing to meet the EAR was 55.5 to 71.3% (18, 27).	N/A	N/A
	Vitamin D	AI: 5 μg/day Serum levels were used as a proxy for intake. 22.9–31.1% of women showed mild to severe deficiency (18).	N/A	Anxiety and depression (≥ 50,000 IU/week) (83) Cancer (high vs. low blood levels; 1,200 to 8,000 IU/day after cancer diagnosis) (84–88) CVD (high vs. low blood levels; 1,000 to 7142.9 IU/day) (89–92) Fertility and pre-pregnancy nutrition (1,000 IU/day; 50,000 IU/week; sufficient vs. insufficient/deficient status) (95, 96) Metabolic health (1,000 to 7142.9 IU/day; 59,000 IU periodically) (92, 93)
	Selenium	EAR: 50 μg/day Mean intake: 47.1–52.3 μg/day 43.8 to 71.7% of NZ women failed to meet the EAR (27).	N/A	N/A
	Folate	N/A	The SDT for dietary folate equivalents is 300–600 μg; an additional 100–400 μg over current intakes (3).	Anxiety and depression (0.5 to 10 mg/day folic acid; 15 mg/day L-methylfolate) (68) Cancer (high vs. low intake; 20 to 30 mg/day folic acid) (69–71) CVD (0.8 mg folic acid) (72) Fertility and pre-pregnancy nutrition (<0.4 to <5 mg/day folic acid) (11, 97)
	Omega-3 fatty acids	N/A	The SDT is 430 mg (3, 61).	Anxiety and depression (all doses, but ≥2000 mg/day better than <2000 mg/day) (73) Cancer (0.51 to 2.2 g/day EPA and 0.24 to 0.92 g/day DHA; high vs. low blood levels DPA or DHA) (74, 75) CVD (high vs. low blood levels DPA, DHA, EPA; 0.5 to >5 g/day; per 1 g/day increase) (72, 75–82) Fertility and pre-pregnancy nutrition (1 to 2 g/day; high vs. low intake) (98) Metabolic health (high vs. low blood markers of omega-3 intake) (75)
Pregnant or lactating females 19–45 y	Vitamin D	AI: 5 μg/day Mean intake: 1.2–4.4 μg/day Mean intake below the EAR in NZ women, and 42% show deficient serum levels (99–102).	N/A	Healthy foetal/infant growth and development (800 to 7142.9 IU/day; each 25 mmol increase in blood status; deficiency/insufficiency vs. sufficiency; high vs. low blood status) (96, 103–111) Healthy maternal weight gain (400 to 3571.4 IU/day; deficiency/insufficiency vs. sufficiency) (107, 112–118) Maternal mental health (blood vitamin D status of 90–110 nmoL/L; 400 to 6,000 IU/day) (119, 120)
Peri or post-menopausal females >45–60 y	Dietary fibre	AI: 25 g/day Mean intake: 18.1–21 g/day) 50^th^ percentile of intake was below the AI for AUS (18) and NZ (27) women.	The SDT is 28 g (3, 61).	Cancer (high vs. low intake) (62, 63) CVD (high vs. low intake; 10 g/day increase; 3–30 g/day) (64–66) Metabolic health (3–20 g/day) (66)
	Calcium	EAR: 1100 mg/day Mean intake: 741–775 mg/day 88.2 to 91.2% of women failed to meet the EAR (18, 27)	N/A	Bone health (≥ 700 mg/day; optimal intake 1,200 mg/day) (121)
	Vitamin D	AI: 5–10 μg/day Serum levels used as proxy for intake. 21.6% of AUS women showed mild to severe vitamin D deficiency (18).	N/A	Anxiety and depression (≥ 50,000 IU/week) (83) Bone health (Low vs. high serum status; < 400 IU/day (combined with calcium) (122, 123) Cancer (high vs. low blood levels; 1,200 to 8,000 IU/day after cancer diagnosis) (84–88) CVD (high vs. low blood levels; 1,000 to 7142.9 IU/day) (89–92) Metabolic health (1,000 to 7142.9 IU/day; 59,000 IU periodically) (92, 93)
	Omega-3 fatty acids	N/A	The SDT is 430 mg (3, 61).	Anxiety and depression (all doses, but ≥2000 mg/day better than <2000 mg/day) (73) Cancer (0.51 to 2.2 g/day EPA and 0.24 to 0.92 g/day DHA; high vs. low blood levels DPA or DHA) (74, 75) CVD (high vs. low blood levels DPA, DHA, EPA; 0.5 to >5 g/day; per 1 g/day increase) (72, 75–82) Metabolic health (High vs. low blood markers of omega-3 intake) (75)
	Folate	N/A	The SDT s 300–600 μg; an additional 100–400 μg over current intakes (3).	Anxiety and depression (0.5 to 10 mg/day folic acid; 15 mg/day L-methylfolate) (68) Cancer (high vs. low intake; 20 to 30 mg/day folic acid) (69–71) CVD (0.8 mg folic acid) (72)
Older adults (males and females) >60 y	Vitamin D	AI: 15 μg/day Serum levels were used as a proxy for intake. 20.2% of older AUS adults showed mild to severe deficiency (18).	N/A	Cognitive function (deficiency) (124) Physical independence (500–1,600 IU/day; 2,500–5,000 IU/week (with protein or exercise); high vs. low serum status; 400–60,000 IU/day (with insufficient serum status) (125–132) CVD (high vs. low blood levels; 1,000 to 7142.9 IU/day) (89–92) Metabolic health (1,000 to 7142.9 IU/day; 59,000 IU periodically) (92, 93)
	Calcium	EAR: 1100 mg/day Mean intake: 690–785 mg/day 86 to 94.3% AUS and NZ older adults failed to meet the EAR (18, 27).	N/A	N/A
	Magnesium	EAR: 265–330 mg/day Mean intake: 274–326 mg/day 48.5 to 63.9% AUS older adults did not meet the EAR (18).	Magnesium requirements should be updated to account factors that can affect the need for magnesium (56).	N/A
	Selenium	AI: 50–60 μg/day Mean intake: 41.6–56.9 μg/day 63.8 to 89.7% NZ older adults failed to meet the EAR (27).	N/A	N/A
	Zinc	EAR: 6.5–12 mg/day Mean intake: 7.9–9.8 mg/day 28.3 to 78.5% NZ older adults failed to meet the EAR (27).	US requirement data may overestimate zinc absorption by 13 to 15% (60); a similar overestimation may be relevant to AUS/NZ.	N/A
	Omega-3 fatty acids	N/A	The SDT is 610 mg for men and 430 mg for women (3, 61).	Physical independence (0.23 to 5 g/day EPA + DHA; 1.35 to 14 g/day ALA; 1.3 to 5 g/day total fatty acids) (133–135) Cognitive function/decline (180 to 2000 mg/day DHA; 40 to 1,080 mg per day EPA) (136–138) CVD (high vs. low blood levels DPA, DHA, EPA; 0.5 to >5 g/day; per 1 g/day increase) (72, 75–82) Metabolic health (high vs. low blood markers of omega-3 intake) (75)

AI, Adequate Intake; AUS, Australia; CVD, cardiovascular disease; DHA, docosahexanoic acid; DPA, docosapentanoic acid; EAR, estimated average requirement; EPA, eicosapentanoic acid; NZ, New Zealand; SDT, Suggested Dietary Target.

When nutrient scoring was pooled and applied at the population level, the top 25% of nutrients were, in order of priority, vitamin D, calcium, omega-3 fatty acids, folate, dietary fibre, and magnesium (Figure 3B). These nutrients represent those with the highest levels of inadequacy and increased needs, and an association with health priorities across the total population. The final scoring for each nutrient across each of the three scoring domains was shown in grey (inadequacy), red (increased needs) and blue (health priority). While vitamin D, omega-3 fatty acids, dietary fibre, and folate achieved their highest scores for association with health priorities, calcium and magnesium ranked the highest for inadequacy. Magnesium also ranked the highest for increased needs. Four of the highest priority nutrients at the population level were also those with the highest prevalence of priority across individual demographic groups; these were vitamin D, calcium, omega-3 fatty acids, and magnesium.

## Discussion

The research presented here has highlighted the priority nutrients, or those with the highest potential to address the double burden of malnutrition and diet-related disease in Australia and New Zealand, at both the demographic group and population-level. Nutritional inadequacies (relative to current NRVs) were prevalent across all demographic groups, ranging from a minimum of 7 (children) to 16 (males and older adults), highlighting an important gap in intake vs. needs. While the number and specifics of priority nutrients varied between demographic groups, there was notable consistency in the nutrients found to be of highest importance across the demographic groups. Furthermore, this consistency was maintained when priority nutrient scoring was applied at the population level, with four nutrients featuring as the highest priority regardless of scoring method; these were vitamin D, calcium, omega-3 fatty acids, and magnesium. Dietary fibre and folate were also priority nutrients at the population level. The methodology adopted to uncover the highest priority nutrients was evidence-based and systematic and provides a novel approach that can be replicated for application on a global scale.

The highest priority nutrients at the population level arose due to not only overt inadequacy, but increased needs and health priorities, addressing both sides of the double burden of disease. While priority for each nutrient was based on the combination of scoring within each one of these domains, each priority nutrient showed variation in terms of which domain made the largest contribution to the total score. This suggests that the two major diet-related issues of malnutrition and diet-related disease are both independent and integrated; a nutrient inadequacy can be an issue due to its necessity for normal physiology, but might also contribute, either directly or indirectly, to the development of one or more diet-related diseases. For example, calcium had a relatively low number of health priority associations (4: bone growth in childhood and adolescence, bone maintenance in peri-menopausal and menopausal women, and healthy weight gain during pregnancy), and intakes associated with health benefits in these health priorities (120–1,200 mg/day in pregnancy) were comparable to recommended values. However, dietary inadequacy was high, with up to 94% not meeting the EAR, thus likely to exacerbate calcium-related disease prevalence. On the other hand, even if met, recommended intakes may not support optimal health or the prevention of diet-related disease. This is evidenced by the development of SDTs for some nutrients (3). For example, while no dietary inadequacy was identified for the omega-3 fatty acids, this nutrient had one of the highest scores for association with health priorities, has an SDT, showing that increased intakes are likely necessary to optimally support health. Regardless of the underlying mechanism, increased intakes are needed for each one of the priority nutrients, and a focus on these nutrients in public nutrition policy and messaging, including the promotion of foods supplying these nutrients, is needed. While previous efforts to uncover priority nutrients have been employed, including nutrition surveys and the development of SDTs (3), our research and scoring matrix integrates three key domains (inadequate intake, evidence for increased needs, and association with health priorities) related to nutrient intake and health to pinpoint the nutrients that may represent the biggest bottlenecks for public nutrition and disease reduction.

The specific priority nutrients identified for each demographic group contribute key information to the recommendations of policy makers, as well as health organisations and professionals, including GPs, nurses, and paediatric dietitians, to name a few. For example, Vitamin D emerged as a priority nutrient for all demographic groups. This finding is consistent with recent dietary intake and blood status data suggesting that the prevalence of vitamin D deficiency and insufficiency are critically high and potentially increasing, with a call for changes in policy to address this (17). However, vitamin D was the sole priority nutrient for pregnant and lactating women, indicating that a targeted focus within this demographic group may be warranted. Similarly, each demographic group was characterised by a unique set of priority nutrients, suggesting that these nutrients require specific attention to optimally support health within that group. For example, five (zinc, folate, omeg-3 fatty acids, vitamin D, and magnesium) of the six priority nutrients identified for males were associated with supporting the health priority of anxiety and depression in this demographic group. Despite identification as a health priority, anxiety and depression have been suggested to be largely overlooked and/or under-reported in adult males (139, 140), accentuating that the priority placement of these nutrients is warranted and has the potential to address the specific needs of each demographic group.

It is important to highlight that 22 nutrients were identified as having inadequate intake in at least one demographic group, despite Australia and New Zealand being developed countries. These nutrients included vitamin A, iron, and iodine, the primary nutrients of concern for deficiency on a global scale (141). Alongside a clear role in health and disease prevention, nutrient and food group inadequacies have significant economic implications, including both health-care costs and losses in productivity. A recent systematic review to assess the cost vs. benefit of folic acid fortification for the prevention of neural tube defects identified a return of 17.5 monetary units for each monetary unit spent, with doses above 300 μg/100 g showing the highest benefit (142). Research on the cost of iron deficiency for 10 developing countries suggested that the median value of annual physical productivity losses was around $2.32 *per capita*, or 0.57% of GDP, with median total losses (physical and cognitive combined) at $16.78 *per capita*, 4.05% of GDP (143). Not surprisingly, there is a cost benefit of addressing deficiency; it has been estimated that addressing micronutrient (iodine, iron, or vitamin A) deficiency via supplementation or fortification efforts produces an economic gain, of 4 to 37 US dollars per disability adjusted life-year (144). In America, $12.7 billion in direct health care costs was the estimated saving if all adults were to consume the US Food and Drug Administration Daily Reference Value for dietary fibre of 25 grams per day (145). Research to determine the cost benefit of addressing priority nutrients in Australia and other developed countries is needed.

In addition to a focus on specific priority nutrients, diet- and food-based recommendations can help to support their intake. Table 6 shows the major dietary sources for each of the population-level priority nutrients (3, 146); while folate and dietary fibre feature primarily in plant-based sources, the major dietary contributors of vitamin D and calcium are animal foods, while magnesium can be found in both. Despite the omega-3 fatty acids also being provided by both plant and animal foods, the forms are not synonymous and have different roles within the body. Further, while ALA (found in nuts and seeds) can be converted into DHA and EPA (found in fatty fish), the efficiency of this conversion is both low and varies among individuals, and much of the literature regarding health associations was focused on DHA, DPA, and EPA. Some foods also provide more than one priority nutrient, for example, fatty fish provide both omega-3 fatty acids and vitamin D, and dairy milk provides both calcium and magnesium. The identification and specific targeting of key foods providing priority nutrients may serve as a useful and effective way to address the lowest-hanging fruit for both malnutrition and diet-related disease burdens.

**Table 6:** Primary dietary sources for each population-level priority nutrient.

Nutrient	Primary dietary sources
Vitamin D	Fatty fish such as salmon, herring and mackerel, and eggs In AUS, fortification is mandated for edible oil spreads (table margarine) and voluntary for modified and skim milks, powdered milk, yoghurts and table confections and cheese; fortified margarine is a major source in Australia. In New Zealand, voluntary fortification of margarine, fat spreads and their reduced fat counterparts has been permitted. It is also permitted to add vitamin D to dried milk, dried skim milk and non-fat milk solids, skim milk and reduced fat cows’ milk, legume beverages and ‘food’ drinks.
Calcium	Primary source is dairy milk and dairy milk-based foods. Smaller amounts in bony fish, legumes and certain nuts, fortified plant-based beverages, and breakfast cereals.
Omega-3 fatty acids	ALA is found in legumes, canola oils and margarines, linseed oils and products, certain nuts such as walnuts, and in small amounts in leafy vegetables; legumes also contribute some. EPA, DHA, and DPA predominantly in oily fish such as mackerel, herrings, sardines, salmon and tuna, and other seafood.
Dietary fibre	Plant-based foods and food products, including vegetables, fruits, legumes and pulses, nuts and seeds, and grains.
Magnesium	Widely distributed in both plant and animal foods. Richest sources are most green vegetables, legumes, peas, beans and nuts, plus shellfish and spices. Milk and milk products. Most unrefined cereals are reasonable sources, but highly refined flours, tubers, fruits, oils and fats contribute little.
Folate	Main sources in Australia and New Zealand are cereals/grains, cereal products and dishes based on cereals, and vegetables and legumes. Fruit provides smaller amount; orange juice provides a notable contribution due to the recent introduction of fortification with folate.

Primary dietary sources based on information provided within the NHMRC Nutrient Reference Values (3) and Australian Dietary Guidelines (Eat for Health Educator’s Guide) (146). ALA, alpha linolenic acid; DHA, docosahexanoic acid; DPA, docosopentanoic acid; EPA, eicosapentanoic acid.

In theory, a balanced diet containing all core food groups, as well as fatty fish, should be able to provide all priority nutrients. However, there is a current trend towards plant-based diets to support health and sustainability, coupled with an increased use of and/or demand for plant-based meat and milk alternatives (147, 148). As these foods are often matched (to their animal-based counterpart) for use but not nutritional composition and/or adequacy (141, 149), these dietary patterns may lead to further increases in the inadequacy level of some of these key nutrients. This pattern may be of even greater importance given that the dietary intake data obtained for the current research was based on the most recent survey data available, now up to 20 years old; the intake of some nutrients may have declined even further with these shifts in dietary patterns. Concern has been expressed regarding the use of plant-based milk alternatives as a substitute for dairy milk, except for fortified soy milk, due to nutritional inequivalence (148, 150). In children, this substitution has been suggested to result in severe consequences for metabolic health (147). As calcium has been identified as having inadequate intakes in both Australia and New Zealand, for which the major dietary source is dairy products (Table 6) an increased shift from dairy milk to plant-based milks may further exacerbate these inadequacies (16, 18). Further, the dietary modelling used to develop the most recent Australian Dietary Guidelines produced dietary patterns that supported the intake of all essential nutrients except for vitamin D (151). These issues need to be addressed and taken seriously, particularly for dietary guidelines. There is a need to balance recommendations to meet nutrient adequacy with dietary shifts in plant-based eating for health as well as for environment for all demographic groups.

Rather than existing and acting in isolation, each dietary nutrient interacts and can act synergistically with other dietary nutrients, both within foods and inside the body following digestion and absorption (152, 153). While outside of the scope of this research, inadequacies in priority nutrients may impact the provision and activity of other nutrients. These interactions may also be involved in the associations identified for priority nutrients and selected health priorities. These factors are important considerations for future research as well as recommendations made by dietary guidelines.

This research has a number of strengths and limitations. First, the holistic and integrated approach of identifying priority nutrients is novel in its ability to determine the priority nutritional targets for both individual demographic groups, as well as the total population, addressing both sides of the nutritional double burden: malnutrition and diet-related disease. As current efforts focus on nutrient intake or health outcomes independently, they lack the ability to target both at the same time. Second, only high-level evidence [based on the NHMRC framework (42)] in nutrition was collected to ensure robustness in the study design and support the conclusions made. Third, the methodology described can be applied to other countries, to highlight nutrients that have importance on a global scale and allowing for comparisons in nutrient inadequacy and nutrient prioritisation. While some nutrients [such as vitamin D (7)] show widespread deficiency and many diet-related diseases are common throughout developed countries (such as cardiometabolic issues and cancer), each country is likely to be characterised by differing degrees of inadequacy for each nutrient, as well as specific nutrient inadequacies, depending on the food environment. In addition, leading causes of morbidity and mortality may differ between countries; for example, cardiovascular complications are a leading cause of maternal mortality in the US (154) but were not identified as such in Australia and New Zealand. Application of the methodology to additional countries and subsequent comparisons are warranted.

Although the most recent national nutrition survey data were utilised, the major limitation of the findings is the age of the most recent dietary intake data, collected in 2011–2012 for Australia, 2007–2008 for New Zealand adults, and 2003 for New Zealand children; and it is likely that dietary patterns have evolved and shifted since this time, indicating that a repeat of this research when new data become available will be essential. Regardless, the findings provide a proof-of-concept for the novel methodology presented; furthermore, given that many of the nutrient inadequacies identified are mimicked by current evidence worldwide (17, 155, 156), these findings remain relevant despite the age of the included data. In addition, while the level of nutrient inadequacy was a major aspect of the methodology, nutrient adequacy alone is not sufficient to prevent disease; there are many factors involved, including (but not limited to) physical activity, smoking and alcohol, social connection, and mental health and wellbeing. However, nutrient adequacy is a key part of any solution, and the current research was focused on nutrition. The methodology for the selection of health priorities was designed to identify disease categories regarded by authorities as being most pertinent to public health in Australia and New Zealand. While robust and specific, this approach does not consider individual diseases of significance within each disease category, nor additional health outcomes showing increasing significance. For example, a high prevalence of vitamin D deficiency was identified in patients with cancer sub-category of neuroendocrine neoplasms (157), and gastrointestinal diseases such as inflammatory bowel disease are both increasing in prevalence and involve malnutrition (158). Further iterations of the methodology can seek to include such diseases and their nutritional relationships. Finally, relating single nutrients to chronic disease outcomes has shortcomings, due to disease being complex and multifaceted in its causes and presentation. Food context, nutrient interactions, and overall dietary patterns play key roles in health, and should be taken into consideration when interpreting the findings presented here, though the NRVs tend to allow for these complex interactions when setting reference values. The overall opportunity lies in the translation of these priority nutrients to food and diet-based recommendations. In line with this, the study did not address non-nutritive dietary factors, which are additional mediators of disease (such as antioxidants), and should be considered in policies, guidelines, and/or strategies that use diet to address health priorities. Further research to understand the highest priority non-nutrients is required.

## Conclusion

Multi-nutrient inadequacies within Australia and New Zealand were reported across all demographic groups, including nutrients with a direct link to increasing rates of diet-related disease. There is a need to revisit and revise nutrient and food-based recommendations, to ensure adequacy for the population, with a focus on priority nutrients that are more likely to impact health. Efforts to provide education and awareness about priority nutrients, to ensure that the current double burden of malnutrition and diet-related disease is alleviated, rather than exacerbated are warranted. Future research needs to focus on the identification and promotion of foods that contribute to priority nutrient intake, whilst also supporting sustainability efforts and taking into consideration cost. While this research was focused on the Australian and New Zealand populations, the methodology presented can be applied globally, to determine and communicate nutrients for prioritisation and nutrition, a fundamental cornerstone for the reduction of diet-related disease.

## Data availability statement

Publicly available datasets were analyzed in this study. This data can be found at: National Nutrition and Physical Activity Survey 2011–2012, https://www.abs.gov.au/statistics/health/health-conditions-and-risks/australian-health-survey-nutrition-first-results-foods-and-nutrients/latest-release and New Zealand Adult Nutrition Survey 2008–2009 and Children’s Nutrition Survey 2002, https://www.health.govt.nz/nz-health-statistics/surveys/past-surveys/nutrition-survey.

## Ethics statement

Ethical approval was not required for the study involving humans in accordance with the local legislation and institutional requirements. Written informed consent to participate in this study was not required from the participants or the participants’ legal guardians/next of kin in accordance with the national legislation and the institutional requirements.

## Author contributions

CS: Conceptualization, Data curation, Formal analysis, Investigation, Methodology, Writing – original draft, Writing – review & editing. TC: Conceptualization, Methodology, Writing – review & editing. EB: Writing – review & editing. SM: Conceptualization, Methodology, Writing – review & editing. FF-M: Conceptualization, Funding acquisition, Methodology, Writing – review & editing.
