# Supplementary material for: Priority nutrients to address malnutrition and diet-related diseases in Australia and New Zealand
Source: Front Nutr. 2024 Mar 13;11:1370550. doi: 10.3389/fnut.2024.1370550 (PMC10966131; doi:10.3389/fnut.2024.1370550)
Supplement: Supplementary file 1 [file Data_Sheet_1.docx]

Supplementary Material

**Supplementary Table 1.** Search strategy to identify associations between each of the nutrients with each identified health priority for each demographic group.

| **Component of search** | **Search strategy** |
| --- | --- |
| **Nutrients** | ((((((((((((((((((((((((((((((((((((((((((((((((((("omega 3"[Title/Abstract]) OR ("omega 6"[Title/Abstract])) OR ("Dietary fibre"[Title/Abstract])) OR ("dietary fiber"[Title/Abstract])) OR (Docosapentaenoic[Title/Abstract])) OR (Eicosapentaenoic[Title/Abstract])) OR (Docosahexaenoic[Title/Abstract])) OR (DPA[Title/Abstract])) OR (EPA[Title/Abstract])) OR (DHA[Title/Abstract])) OR (linolenic[Title/Abstract])) OR (linoleic[Title/Abstract])) OR (protein[Title/Abstract])) OR (tocopherol[Title/Abstract])) OR (retinol[Title/Abstract])) OR (pyridoxine[Title/Abstract])) OR (cobalamin[Title/Abstract])) OR (niacin[Title/Abstract])) OR (riboflavin[Title/Abstract])) OR (thiamin[Title/Abstract])) OR (choline[Title/Abstract])) OR (biotin[Title/Abstract])) OR (pantothenic[Title/Abstract])) OR ("folic acid"[Title/Abstract])) OR (folate[Title/Abstract])) OR (vitamin[Title/Abstract])) OR (zinc[Title/Abstract])) OR (sodium[Title/Abstract])) OR (selenium[Title/Abstract])) OR (potassium[Title/Abstract])) OR (phosphorus[Title/Abstract])) OR (molybdenum[Title/Abstract])) OR (manganese[Title/Abstract])) OR (magnesium[Title/Abstract])) OR (iodine[Title/Abstract])) OR (fluoride[Title/Abstract])) OR (copper[Title/Abstract])) OR (chromium[Title/Abstract])) OR (Calcium[Title/Abstract])) OR ("dietary fiber"[MeSH Terms])) OR (fatty acids, unsaturated[MeSH Terms])) OR (fatty acids, omega 6[MeSH Terms])) OR (fatty acids, omega 3[MeSH Terms])) OR ("fatty acids, essential"[MeSH Terms])) OR ("diet therapy"[MeSH Terms])) OR ("phosphorus"[MeSH Terms])) OR ("trace elements"[MeSH Terms])) OR ("micronutrients"[MeSH Terms])) OR ("avitaminosis"[MeSH Terms])) OR ("avitaminosis"[MeSH Terms])) OR ("nutrients"[MeSH Terms])) |
| ***AND* Study Types** | ((("meta analysis"[Publication Type]) OR ("guideline"[Publication Type])) OR ("systematic review"[Publication Type])) |
| ***AND* Anxiety and depression** | (((((((("mental health"[MeSH Terms]) OR ("anxiety disorders"[MeSH Terms])) OR ("disruptive, impulse control, and conduct disorders"[MeSH Terms])) OR (mood disorders[MeSH Terms])) OR (Depression[Title/Abstract])) OR (Anxiety[Title/Abstract])) OR (anxious[Title/Abstract])) OR (depressive[Title/Abstract])) OR ("mental health"[Title/Abstract]) |
| ***OR* Infectious immunity** | (((("immunity"[MeSH Terms]) OR ("infections"[MeSH Terms])) OR (immunity[Title/Abstract])) OR (immune[Title/Abstract])) OR (infection*[Title/Abstract]) **AND**  (((((("child"[MeSH Terms]) OR ("adolescent"[MeSH Terms])) OR (child*[Title/Abstract])) OR (teen*[Title/Abstract])) OR (adolescent*[Title/Abstract])) OR (pediatric*[Title/Abstract])) OR (paediatric*[Title/Abstract]) |
| ***OR* Bone health and bone mass acquisition** | (((((((("bone density"[MeSH Terms]) OR ("bone and bones"[MeSH Terms])) OR (bone diseases, metabolic[MeSH Terms])) OR ("bone density"[Title/Abstract])) OR ("fractures, bone"[MeSH Terms])) OR (fracture*[Title/Abstract])) OR (osteoporo*[Title/Abstract])) OR (osteopeni*[Title/Abstract])) OR ("musculoskeletal development"[MeSH Terms]) **AND**  ((((((("child"[MeSH Terms]) OR ("adolescent"[MeSH Terms])) OR (child*[Title/Abstract])) OR (teen*[Title/Abstract])) OR (adolescen*[Title/Abstract])) OR (pediatric*[Title/Abstract])) OR (paediatric*[Title/Abstract])) OR (((("menopause"[MeSH Terms]) OR ("perimenopause"[MeSH Terms])) OR (perimenopausal[Title/Abstract])) OR (menopaus*[Title/Abstract])) |
| ***OR* Muscle and sexual organ growth and development** | ((((((("musculoskeletal development"[MeSH Terms]) OR ("puberty"[MeSH Terms])) OR ("genitalia"[MeSH Terms])) OR (puberty[Title/Abstract])) OR (skeletal[Title/Abstract])) OR (muscle[Title/Abstract])) OR ("sex organ"[Title/Abstract])) OR ("sexual organ"[Title/Abstract]) **AND**  ((((("adolescent"[MeSH Terms]) OR (adolescen*[Title/Abstract]))) OR (adolescen*[Title/Abstract])) OR (pediatric*[Title/Abstract])) OR (paediatric*[Title/Abstract]) |
| ***OR* Cognitive development** | ((((((("cognition"[MeSH Terms]) OR ("intelligence"[MeSH Terms])) OR ("executive function"[MeSH Terms])) OR (brain[Title/Abstract])) OR (cognitive[Title/Abstract])) OR (cognition[Title/Abstract])) OR (IQ[Title/Abstract])) OR (intelligence[Title/Abstract]) **AND**  ((((((("child"[MeSH Terms]) OR ("adolescent"[MeSH Terms])) OR (child*[Title/Abstract])) OR (teen*[Title/Abstract])) OR (adolescen*[Title/Abstract])) OR (pediatric*[Title/Abstract])) OR (paediatric*[Title/Abstract])) OR (((("menopause"[MeSH Terms]) OR ("perimenopause"[MeSH Terms])) OR (perimenopausal[Title/Abstract])) OR (menopaus*[Title/Abstract])) |
| ***OR* Cancer** | ((((((("neoplasms"[MeSH Terms]) OR (cancer[Title/Abstract])) OR (tumour[Title/Abstract])) OR (tumor[Title/Abstract])) OR (lymphoma[Title/Abstract])) OR (myeloma[Title/Abstract])) OR (leukemia[Title/Abstract])) OR (oncolog*[Title/Abstract]) |
| ***OR* Cardiovascular disease** | ((((((((("heart disease risk factors"[MeSH Terms]) OR ("heart diseases"[MeSH Terms])) OR ("cardiovascular diseases"[MeSH Terms])) OR (CVD[Title/Abstract])) OR (CHD[Title/Abstract])) OR (IHD[Title/Abstract])) OR (cardiovascular[Title/Abstract])) OR (coronary[Title/Abstract])) OR ("heart disease"[Title/Abstract])) OR (stroke[Title/Abstract]) |
| ***OR* Metabolic health** | (((((((((("obesity"[MeSH Terms]) OR ("metabolic syndrome"[MeSH Terms])) OR ("insulin resistance"[MeSH Terms])) OR ("diabetes mellitus, type 2"[MeSH Terms])) OR ("metabolic diseases"[MeSH Terms])) OR (T2DM[Title/Abstract])) OR (T2D[Title/Abstract])) OR (“type 2 diabetes”[Title/Abstract])) OR (“metabolic syndrome”[Title/Abstract])) OR (“insulin resistance”[Title/Abstract])) OR (“blood glucose”[Title/Abstract])) OR ("hyperglycemia"[Title/Abstract])) OR (hyperglycaemia[Title/Abstract]) OR (insulin[Title/Abstract])) OR (hyperinsulinemia*[Title/Abstract])) OR (hyperinsulinaemia*[Title/Abstract])) OR (bodyweight[Title/Abstract])) OR (“waist circumference”Title/Abstract])) |
| ***OR* Fertility and pre-pregnancy nutrition** | ((("fertility"[MeSH Terms]) OR ("pregnancy rate"[MeSH Terms])) OR (Fertility[Title/Abstract])) OR ("pre pregnancy"[Title/Abstract]) |
| ***OR* Maternal weight gain and foetal development** | **(((("fetal blood"[MeSH Terms]) OR ("pregnancy complications"[MeSH Terms])) OR ("pregnancy outcome"[MeSH Terms])) OR ("gestational weight gain"[MeSH Terms])) OR ("fetal weight"[MeSH Terms])** |
| ***OR* Cognitive function or decline** | ((((("dementia"[MeSH Terms]) OR ("cognition"[MeSH Terms])) OR (dementia[Title/Abstract])) OR (alzheimer*[Title/Abstract])) OR (cognition[Title/Abstract])) OR (cognitive[Title/Abstract]) AND  ((((("aged"[MeSH Terms]) OR ("aged, 80 and over"[MeSH Terms])) OR (elderly[Title/Abstract])) OR (old[Title/Abstract])) OR (older[Title/Abstract])) OR (geriatric[Title/Abstract]) |
| ***OR* Physical independence** | (((((((((((((((((((("frailty"[MeSH Terms]) OR ("malnutrition"[MeSH Terms])) OR ("sarcopenia"[MeSH Terms])) OR (frailty[Title/Abstract])) OR (malnutrition[Title/Abstract])) OR (sarcopenic[Title/Abstract])) OR (sarcopenia[Title/Abstract])) OR ("muscle wasting"[Title/Abstract])) OR ("muscle maintenance"[Title/Abstract])) OR (ADLs[Title/Abstract])) OR (ADL[Title/Abstract])) OR ("physical function"[Title/Abstract])) OR (independence[Title/Abstract])) OR (falls[Title/Abstract])) OR (independent living[MeSH Terms])) OR ("functional status"[MeSH Terms])) OR ("accidental falls"[MeSH Terms])) OR ("musculoskeletal development"[MeSH Terms])) OR ("activities of daily living"[MeSH Terms])) OR ("institutionalization"[MeSH Terms])) OR ("physical and rehabilitation medicine"[MeSH Terms]) AND (((((("aged"[MeSH Terms]) OR ("aged, 80 and over"[MeSH Terms])) OR (elderly[Title/Abstract])) OR (old[Title/Abstract])) OR (older[Title/Abstract])) OR (geriatric[Title/Abstract])) AND  (((((("dementia"[MeSH Terms]) OR ("cognition"[MeSH Terms])) OR (dementia[Title/Abstract])) OR (alzheimer*[Title/Abstract])) OR (cognition[Title/Abstract])) OR (cognitive[Title/Abstract])) |

**Supplementary Table 2.** Full list of health priorities identified for each demographic group under each category (leading causes of death, physical morbidity, mental or cognitive ill-health, and other) and final health priorities selected for literature review.

| **Demographic group** | **Leading causes of death** | **Leading causes of physical morbidity** | **Leading causes of mental or cognitive ill-health** | **Other^1^** | **Final selected health priorities** |
| --- | --- | --- | --- | --- | --- |
| Children (male and female) aged 4-11 years | - Influenza/pneumonia | - Asthma | - Anxiety disorders | - Growth and development (bone) - Growth and development (cognitive) | - Bone mass acquisition - Cognitive development - Infectious immunity |
| Teenagers (male and female) aged 12-18 years | None | - Chronic pain - Asthma | - Anxiety disorders - Depressive disorders | - Growth and development (bone) - Growth and development (cognitive) - Pubertal maturation | - Anxiety and depression - Cognitive development - Growth and development focusing on bone and muscle - Maturation focusing puberty/sexual organ development |
| Males aged 19-60 years | - CVD - Cancer - Liver disease - Diabetes | - Chronic pain - Asthma - Overweight/obesity | - Anxiety disorders - Depressive disorders | - Metabolic syndrome - Sexual function | - Anxiety and depression - Cancer - CVD - Metabolic disorders |
| Females aged 19-45 years | - Cancer - CVD - Liver disease | - Asthma - Chronic pain - OA - Overweight/obesity | - Anxiety disorders - Depressive disorders | - Child-bearing potential | - Anxiety and depression - Cancer - CVD - Fertility and pre-pregnancy nutrition - Metabolic disorders |
| Pregnant or lactating females aged 19-45 years | None | - Gestational diabetes - Hypertension | None | - Healthy foetal/infant growth and development - Healthy maternal weight gain - Maternal mental health - Healthy milk production | - Healthy foetal/infant growth and development. - Healthy maternal weight gain. - Maternal mental health. |
| Peri-menopausal or menopausal females aged >45-60 years | - Cancer - CVD - Liver disease - Diabetes | - Chronic pain and arthritis - OA - Asthma - Overweight/obesity | - Anxiety disorders - Depressive disorders | - Vasomotor menopausal symptoms - Prevention of bone density loss | - Anxiety and depression - Bone health - Cancer - CVD - Metabolic disorders |
| Older adults (males and females) aged >60 years | - CVD - Cancer - Lung disease - Influenza/ pneumonia | - Arthritis - Asthma - Osteoarthritis - Overweight/obesity - T2DM - Hypertension | - Dementia - Anxiety disorders - Depressive disorders | - Physical independence | - Cognitive function / decline - CVD - Physical independence (i.e., muscle and bone maintenance, falls risk) - Metabolic disorders |

CVD, cardiovascular disease; IHD, ischemic heart disease; OA, osteoarthritis. 1. Identified by the panel of health care professionals as per the methodology.

**Supplementary Table 3.** Nutrients identified for each demographic as having an inadequate intake.

| **Demographic group** | **Nutrient & level of inadequacy** |
| --- | --- |
| Children (male and female) aged 4-11 years | - *Dietary fibre:* 50^th^ percentile of intake (16.4 to 19.4 g/day) was below the AI for New Zealand children (1). - *Omega-6 fatty acids:* 50^th^ percentile of intake for Australian 4 to 8 year old boys and girls (6 to 7 g/day) and 9 to 13 year old boys (9 g/day) was below the AI (2). - *Choline:* Mean intake in Australian children (203.4 to 244.2 mg/day) was below the AI (3). - *Potassium:* 50^th^ percentile of intake in Australian children (1983 to 2706 mg/day) was below the AI (2); 50^th^ percentile of intake in New Zealand girls (2272 mg/day) was below the AI (1). - *Calcium:* 45.5% of Australian boys and 54.1% Australian girls did not meet the EAR (2). - *Fluoride:* Estimated mean intake in New Zealand girls (0.38 to 1.03 mg/day) was below the AI (4). - *Vitamin B6:* 20.1% of Australian children aged 9 to 11 years had intakes below the EAR (2). - *Magnesium:* 24.6% of Australian girls did not meet the EAR (2). - *Phosphorus:* 27.6% of Australian girls did not meet the EAR (2). |
| Teenagers (male and female) aged 12-18 years | - *Dietary fibre :* 50^th^ percentile of intake (19 to 22 g/day) was below the AI for Australian teenagers (2); 50^th^ percentile of intake (15.6 to 21.1 g/day) was below the AI for New Zealand teenagers(5). - *Omega-6 fatty acids:* 50^th^ percentile of intake for Australian males (10 g/day) was below the AI (2). - *Vitamin A:* The proportion of Australian and New Zealand teenagers failing to meet the EAR ranged from 26.5% to 37.5% (2, 5). - *Vitamin B6:* The proportion of Australian teenagers failing to meet the EAR ranged from 20.3% to 37.4% (2). - *Choline:* Mean intake in Australian teenagers (229.2 to 275.4 mg/day) was below the AI (3). - *Calcium:* The proportion of Australian teenagers failing to meet the EAR was 67% to 90.3% (2); the proportion of New Zealand teenagers failing to meet the EAR was 59.1% to 87.8% (5). - *Fluoride:* Estimated mean intake in New Zealand males and females (0.73 to 1.89 mg/day) was below the AI (4). - *Iodine:* No deficiency by dietary intake for Australian teenagers (2); 46% of New Zealand teenagers showed a moderate iodine deficiency by urinary analysis (5). - *Magnesium:* Up to 71.6% of Australian teenagers did not meet the EAR (2). - *Potassium:* 50^th^ percentile of intake in Australian and New Zealand teenagers (2239 to 3185 mg/day) was below the AI (2, 5). - *Selenium:* Proportion of New Zealand teenagers failing to meet the EAR was 40.4% to 78.2% (5). - *Zinc:* 27.4% of Australian males failed to meet the EAR (2). - *Thiamin:* 38.3% of New Zealand females did not meet the EAR (5). - *Vitamin E:* 50^th^ percentile of intake (7.5 mg/day) was below the EAR for New Zealand females (5). - *Iron:* 40.1% of Australian females aged 14 to 18 years did not meet the EAR(2); 34.2% of New Zealand females did not meet the EAR (5). - *Phosphorus:* 29.8% of New Zealand females did not meet the EAR (5). |
| Males aged 19-60 years | - *Omega-6 fatty acids:* 50^th^ percentile of intake for Australian men (9 to 11 g/day) was below the AI (2). - *Dietary fibre:* 50^th^ percentile of intake (23 to 24 g/day) was below the AI for Australian men (2); 50^th^ percentile of intake (20.8 to 23.4 g/day) was below the AI for New Zealand men (5). - *Vitamin A:* The proportion of Australian and New Zealand men failing to meet the EAR was up to 30.5% (2, 5). - *Vitamin D:* Serum levels were used as a proxy for vitamin D intake; >20% of Australian men showed mild to severe vitamin D deficiency (2). - *Choline:* Mean intake in Australian men (310.5 mg/day) was below the AI (3). - *Calcium:* The proportion of Australian and New Zealand men failing to meet the EAR was 31.9% to 63% (2, 5). - *Fluoride:* Estimated mean intake in New Zealand men (0.8 to 2.5 mg/day) was below the AI (4). - *Iodine:* 39% to 47% of New Zealand men showed a moderate iodine deficiency by urinary analysis (5). - *Magnesium:* 33% to 46.5% of Australian men did not meet the EAR (2). - *Potassium:* 50^th^ percentile of intake in Australian (3151 to 3271 mg/day) and New Zealand men (3319 to 3724 mg/day) was below the AI (2, 5). - *Selenium:* The proportion of New Zealand men failing to meet the EAR was 29.7% to 46.8% (5). - *Zinc:* The proportion of Australian and New Zealand men failing to meet the EAR was 24.2% to 51.4% (2, 5). - T*hiamin:* 20.5% of New Zealand men aged 51-60 years did not meet the EAR (5). - *Vitamin B6:* 27.5% of New Zealand men aged 51-60 years did not meet the EAR (5); 38.9% of Australian men aged 51-70 years did not meet the EAR (2). - *Pantothenic acid:* The mean intake of 31-50 year old Australian men (5.23 to 5.27 mg/day) was below AI (6). - *Biotin:* The mean intake of 31-50 year old Australian men (22.13 to 23.34 mg/day) was below AI (6). |
| Females aged 19-45 years | - *Dietary fibre:* 50^th^ percentile of intake (19 to 20 g/day) was below the AI for Australian women (2); 50^th^ percentile of intake (17 to 17.9 g/day) was below the AI for New Zealand women (5). - *Thiamin:* The proportion of Australian and New Zealand women failing to meet the EAR was up to 30.1% (2, 5). - *Vitamin B6:* 34% to 38% of Australian women did not meet the EAR (2). - *Vitamin D:* Serum levels were used as a proxy for vitamin D intake; 22.9 to 31.1% of women showed mild to severe vitamin D deficiency (2). - *Biotin:* Mean intake of Australian women (22.13-23.34 mg/day) was below AI (6). - *Choline:* Mean intake in Australian women (247.7 mg/day) was below the AI (3). - *Calcium:* The proportion of Australian and New Zealand women failing to meet the EAR was 52.4% to 71.3% (2, 5). - *Fluoride:* Estimated mean intake in New Zealand women (0.88 to 2.35 mg/day) was below the AI (4). - *Iodine:* 51% to 53% of New Zealand women showed a moderate iodine deficiency by urinary analysis (5). - *Magnesium:* Up to 37.2% of Australian women did not meet the EAR (2). - *Selenium:* The proportion of New Zealand women failing to meet the EAR was 43.8% to 71.7% (5). - *Vitamin B12:* 22.8% of 19-30 year old New Zealand women did not meet the EAR (5). - *Iron:* 37.5% of Australian women did not meet the EAR (2) - *Potassium:* 50^th^ percentile of intake in Australian women aged 19 to 45 years (2405 to 2575 mg/day) and New Zealand women aged 19 to 30 years (2643 mg/day) was below the AI (2, 5). |
| Pregnant or lactating females aged 19-45 years | - *Dietary fibre:* 50^th^ percentile of intake (25.8 g/day) was below the AI for Australian women (7); mean intake (23 to 24 g/day) was below the AI for New Zealand women (8, 9). - *Vitamin B6:* Mean intake (1.5 to 1.9 mg/day) was below the EAR for New Zealand women (8, 9). - *Vitamin D:* In New Zealand women, mean intake (1.2-4.4 µg/day) was below the EAR, and 42% show deficient serum levels (8-11). - *Folate:* 25th percentile of intake for Australian women (406.3 µg/day) did not meet the EAR (7); mean intake (232-418 µg/day) was below EAR for New Zealand women (8-10). - *Choline:* Mean intake in Australian women (151.5 to 253.9 mg/day) was below the AI (3). - *Calcium:* Median intake for Australian women (769.5 mg/day) was below the EAR (7); mixed results for NZ women, with a mean intake both below (822 mg/day) and above (953 mg/day) the EAR (8, 9). - *Iodine:* Median intake (121.8 µg/day) for Australian women was below the EAR (7); mean intake for New Zealand women (61.1 to 80 µg/day) was below the EAR (10). - *Iron:* Median intake (10.8 mg/day) for Australian women was below the EAR (7); mean intake for New Zealand women (13.3 to 16.1 mg/day) was below the EAR (10). - *Potassium:* 25^th^ percentile of intake in Australian women (2531.8 mg/day) was below the AI (7). |
| Peri-menopausal or menopausal females aged >45-60 years | - *Dietary fibre:* 50^th^ percentile of intake (21 g/day) was below the AI for Australian women (2); 50^th^ percentile of intake (18.1 g/day) was below the AI for New Zealand women (5). - *Thiamin:* The proportion of New Zealand women failing to meet the EAR was 20.5% (5). - *Vitamin B6:* 36.6% to 58% of Australian and New Zealand women did not meet the EAR (2, 5). - *Vitamin D:* Serum levels were used as a proxy for vitamin D intake; >20% of women showed mild to severe vitamin D deficiency (2). - *Choline:* Mean intake in Australian women (247.7 mg/day) was below the AI (3). - *Calcium:* The proportion of Australian and New Zealand women failing to meet the EAR was 88.2% to 91.2% (2, 5). - *Fluoride:* Estimated mean intake in New Zealand women (0.88 to 2.35 mg/day) was below the AI (4). - *Iodine:* 48% of New Zealand women showed a moderate iodine deficiency by urinary analysis (5). - *Magnesium:* 30.6% of Australian women did not meet the EAR (2). - *Potassium:* 50^th^ percentile of intake in Australian women (2671 mg/day) was below the AI (2). - *Selenium:* The proportion of New Zealand women failing to meet the EAR was 55% (5). |
| Older adults (males and females) aged >60 years | - *Omega-6 fatty acids:* 50^th^ percentile of intake for Australian older adults (7 to 8 g/day) was below the AI (2). - *Dietary fibre:* 50^th^ percentile of intake (17.5 to 20.4 g/day) was below the AI for New Zealand older adults (5). - *Thiamin:* The proportion of Australian and New Zealand older adults failing to meet the EAR was 21.2% to 27.8% (2, 5). - *Riboflavin:* The proportion of Australian older adults failing to meet the EAR was 20.3% (2). - *Vitamin B6:* 28.8% to 56.7% of Australian and New Zealand older adults did not meet the EAR (2, 5). - *Vitamin D:* Serum levels were used as a proxy for vitamin D intake; >20% of older Australian adults showed mild to severe vitamin D deficiency (2). - *Choline:* Mean intake in Australian older adults (249 to 281.3 mg/day) was below the AI (3). - *Calcium:* The proportion of Australian and New Zealand older adults failing to meet the EAR was 86% to 94.3% (2, 5). - *Fluoride:* Estimated mean intake in New Zealand older adults (0.8 to 2.5 mg/day) was below the AI (4). - *Iodine:* 38% to 45% of New Zealand older adults showed a moderate iodine deficiency by urinary analysis (5). - *Magnesium:* 48.5% to 63.9% of Australian older adults did not meet the EAR (2). - *Potassium:* 50^th^ percentile of intake in Australian (2499 to 2946 mg/day) and New Zealand (2567 to 3138 mg/day) older adults was below the AI (2, 5). - *Selenium:* The proportion of New Zealand older adults failing to meet the EAR was 63.8% to 89.7% (5). - *Zinc:* The proportion of New Zealand older adults failing to meet the EAR was 28.3% to 78.5% (5). - *Vitamin A:* 31.6% of New Zealand older men did not meet the EAR (5). - *Vitamin B12:* 27% of New Zealand older women did not meet the EAR (5). |

AI, adequate intake; EAR, estimated average requirement.

**Supplementary Table 4.** Nutrients identified for each demographic as having increased needs.

| **Demographic group** | **Limited absorption, utilisation, or increased losses** | **Evidence that the NRV should be updated** | **Presence of Suggested Dietary Target** |
| --- | --- | --- | --- |
| Children (male and female) aged 4-11 years | - *Iron*: Increased requirement due to iron accretion during childhood growth (12). | - *Protein*: Data from the IAAO has suggested that requirements may be up to 1.55 g/kg bw/day for children 6-10 years (13), almost double the current RDI (0.87 to 0.94 g/kg bw/day, depending on age and sex). - *Magnesium:* An intake of 133 mg/d for children aged 4-8 years old, above the current EAR of 110 mg/d, needed for growth and adequate BMC (14); magnesium requirements should be updated to account factors that can affect the need for magnesium (15). | N/A |
| Teenagers (male and female) aged 12-18 years | - *Calcium:* Limited absorption coupled with a large increase in the rate of skeletal calcium accretion during the teenage years (12, 16). - *Iron:* Increased requirement due to iron accretion during adolescent growth (12). - *Zinc:* Increased requirement for males due to higher losses in semen combined with possible decreased absorptive capacity (12, 16, 17). | - *Protein:* Reanalysis of NB and IAAO data produced RDIs for adults of 0.99 and 1.2 g/kg/day, respectively (18, 19); 30% higher than the current RDI; the addition of requirements for growth to the increased RDI estimates for adults suggests that the RDI for protein in teenagers may also require update. - *Magnesium:* Magnesium requirements should be updated to account factors that can affect the need for magnesium (15). - *Zinc:* US requirement data may overestimate zinc absorption by 10% (17); a similar overestimation may be relevant to AUS/NZ. | N/A |
| Males aged 19-60 years | - *Choline:* Studies suggest that males utilise choline less efficiently than females, leading to higher requirements (12). - *Zinc:* Zinc requirement is higher for males due to losses in semen and possible decreased absorptive capacity (12, 17). | - *Protein:* Reanalysis of NB and IAAO data produced RDIs for adults of 0.99 and 1.2 g/kg/day, respectively (18, 19); 30% higher than the current RDI. - *Vitamin C:* A dose of 200 mg/day (almost 7 times higher than the EAR (12)) necessary to achieve maximum bioavailability, and plasma and tissue concentrations in adults (20). - *Magnesium:* Magnesium requirements should be updated to account factors that can affect the need for magnesium (15). - *Zinc:* US requirement data may overestimate zinc absorption by 13% (17); a similar overestimation may be relevant to AUS/NZ. | - *Long chain omega-3 fatty acids (DHA + DPA) + EPA):* The SDT is 610 mg for men (12, 21). - *Dietary fibre:* The SDT is 38 g/day for men (12, 21). - *Vitamin A :* The SDT for vitamin A in men is 1500 µg (12, 21). - *Vitamin C:* The SDT for vitamin C in men is 220 mg (12, 21). - *Vitamin E:* The SDT for vitamin E in men is 19 mg (12, 21). - *Folate:* The SDT for dietary folate equivalents is 300-600 µg; this range represents an additional 100-400 µg over current intakes (12). - *Potassium:* The SDT for men is 4700 mg (12, 21). |
| Females aged 19-45 years | - *Iron:* Increased losses in women who menstruate (12, 22, 23). | - *Protein:* Reanalysis of NB and IAAO data produced RDIs for adults of 0.99 and 1.2 g/kg/day, respectively (18, 19); 30% higher than the current RDI. - *Vitamin C:* A dose of 200 mg/day (almost 7 times higher than the EAR (12)) necessary to achieve maximum bioavailability, and plasma and tissue concentrations in adults (20); The daily vitamin C intake needed to prevent collagen-related pathologies is in the range of 75 to 110 mg/day (24). - *Magnesium:* Magnesium requirements should be updated to account factors that can affect the need for magnesium (15). - *Zinc:* US requirement data may overestimate zinc absorption by 15% (17); a similar overestimation may be relevant to AUS/NZ. | - *Long chain omega-3 fatty acids (EPA+DPA+DHA):* The SDT is 430 mg (12, 21). - *Dietary fibre:* The SDT is 28 g (12, 21). - *Vitamins A:* The SDT is 1220 µg (12, 21). - *Vitamin C:* The SDT is 190 mg (12, 21). - *Vitamin E:* The SDT is 14 mg (12, 21). - *Folate:* The SDT for dietary folate equivalents is 300-600 µg; this range represents an additional 100-400 µg over current intakes (12). - *Potassium:* The SDT for women is 4700 mg (12, 21). |
| Pregnant or lactating females aged 19-45 years | - *Protein:* Requirements in pregnancy increase by approximately 33% due to increased weight gain, and by 47% during lactation due to the need for protein provision in breast milk (12). - *Folate:* The need for folate is increased during periods of high cell turnover, such as in foetal development (12, 25). Maximised protection against neural tube defects is also obtained with very high intakes of folate (12, 25). - *Pantothenic acid:* Requirements substantially increase during lactation due to the secretion of pantothenate in breast milk (12). - *Iodine:* Increased losses in the urine during pregnancy (25). - *Iron:* Increased accretion during pregnancy (12). - *Selenium:* There may be reduced availability of selenium in pregnancy due to increased oxidative and inflammatory stress and the role of selenium as an antioxidant (25). - *Zinc:* Women often consume supplemental iron during pregnancy; this may lead to decreased zinc absorption, primarily during lactation (12, 26); however, zinc absorption may increase during pregnancy and lactation (27). | - *Protein:* Reanalysis of NB and IAAO data produced RDIs for adults of 0.99 and 1.2 g/kg/day, respectively (18, 19); 30% higher than the current RDI; addition of needs for pregnancy/lactation is likely to produce requirements above the current RDI. - *Magnesium:* Magnesium requirements should be updated to account factors that can affect the need for magnesium (15). - *Zinc:* US requirement data may overestimate zinc absorption by 15% (17); a similar overestimation may be relevant to AUS/NZ. |  |
| Peri -menopausal or menopausal females aged >45-60 years | - *Calcium:* Decreased absorption combined with increased urinary excretion during the perimenopausal period (12, 28). | - *Protein:* Reanalysis of NB and IAAO data produced RDIs for adults of 0.99 and 1.2 g/kg/day, respectively (18, 19); 30% higher than the current RDI. - *Vitamin C:* A dose of 200 mg/day (almost 7 times higher than the EAR (12)) necessary to achieve maximum bioavailability and plasma and tissue concentrations in adults, suggesting that the RDI for vitamin C should be increased (20). - *Magnesium:* Magnesium requirements should be updated to account factors that can affect the need for magnesium (15). - *Zinc:* US requirement data may overestimate zinc absorption by 15% (17); a similar overestimation may be relevant to AUS/NZ. | - *Long chain omega-3 fatty acids (EPA+DPA+DHA):* The SDT is 430 mg (12, 21). - *Dietary fibre:* The SDT is 28 g (12, 21). - *Vitamins A:* The SDT is 1220 µg (12, 21). - *Vitamin C:* The SDT is 190 mg (12, 21). - *Vitamin E:* The SDT is 14 mg (12, 21). - *Folate::* The SDT for dietary folate equivalents is 300-600 µg; this range represents an additional 100-400 µg over current intakes (12). - *Potassium:* The SDT is 4700 mg (12, 21). |
| Older adults (males and females) aged >60 years | - *Protein:* Older adults may have 25% higher requirements to achieve homeostasis (12), due to anabolic resistance (29) and impaired amino acid absorption/utilisation (30). - *Vitamin B12:* Approximately 30% of older adults require additional vitamin B12 due to decreased absorption (30-32); the NRVs are not increased but it is noted some older adults have elevated requirements (12) or reduced absorptive capacity (30-32). - *Vitamin D:* Increased dietary intake is required to maintain homeostasis in older adults and to maintain bone health, regardless of sun exposure, due to low levels of serum vitamin D at requirements set for younger adults (12, 31). - *Calcium:* Decreased absorption is known to occur during ageing (12). - *Zinc:* Increased risk of zinc malabsorption due to highly prevalent malabsorptive factors during older age, including muscle wasting and polypharmacy (32). | - *Protein:* Experts in the field recommend that the intake for older adults be 1.2 to 2.0 g/kg/day (29). These estimates are 12% to over 200% of the current RDI (12). - *Riboflavin:* Older adults were recognised to have higher requirements based on a high rate of deficiency despite intakes in line with the adequate level for adults (12). - *Vitamin C:* A dose of 200 mg/day (almost 7 times greater than the EAR (12)) necessary to achieve maximum bioavailability, and plasma and tissue concentrations in adults, suggesting that the RDI for vitamin C should be increased (20). - *Magnesium:* Magnesium requirements should be updated to account factors that can affect the need for magnesium (15). - *Zinc:* US requirement data may overestimate zinc absorption by 13-15% (17); a similar overestimation may be relevant to AUS/NZ. | - *Long chain omega-3 fatty acids (EPA+DPA+DHA):* The SDT is 610 mg for men and 430 mg for women (12, 21). - *Dietary fibre:* The SDT is 38 g for men and 28 g for women (12, 21). - *Vitamin A:* The SDT in men and women is 1500 µg and 1220 µg, respectively (12, 21). - *Vitamin C:* The SDT in men and women is 220 mg and 190 mg, respectively (12, 21). - *Vitamin E:* The SDT in men and women is 19 mg and 14 mg, respectively (12, 21). - *Folate:* The SDT for dietary folate equivalents is 300-600 µg; this range represents an additional 100-400 µg over current intakes (12). - *Potassium:* The SDT for both men and women is 4700 mg (12, 21). |

AI, adequate intake; AUS/NZ, Australia/New Zealand; BMC, bone mineral content; EAR, estimated average requirement; IAAO, Indicatory Amino Acid Oxidation method; NB, nitrogen balance; NRV, Nutrient Reference Value; RDI, Recommended Dietary Intake; SDT, Suggested Dietary Target; US, United States (of America).

**Supplementary Table 5.** Nutrients identified as being associated with a health priority for each demographic group.

| **Demographic group** | **Health priority** | **Nutrient** | **Effects** | **Dose** |
| --- | --- | --- | --- | --- |
| Children (male and female) aged 4-11 years | Bone mass acquisition | Vitamin D | ↑ total body BMC (2.6%), lumbar spine BMC (1.7%) (in subjects with low serum vitamin D) (33, 34). | 132 IU/day to 14000 IU/week for at least 3 months |
|  |  | Calcium | ↑ femoral neck BMD (increased 0.02 g/cm2) and hip BMD (0.03 g/cm^2^) (35)  ↑ total body BMC (0.14 g), upper limb BMD (increase of 0.14 g/cm^2^) (36, 37) | Increase in calcium intake by 460 mg/day  300-1200 mg/day |
|  | Cognitive development | Vitamin D | ↑ cognitive function (1.22 IQ points) (38) | 60 to 1000 IU per day |
|  |  | Omega 3 fatty acids | ↑ scores on measures of cognition including learning, decision making and memory (39).  ↑ long-term memory, working memory and problem solving (40).  ↑ treatment of autism, ADHD, and psychosis (41). | ≥450 mg/day long chain omega 3s; increase in blood levels (to >6%)  EPA (from 0 to 720 mg/day), but not DPA (from 0-1200 mg/day)  0.1 to 2.2 g/day omega-3 fatty acids |
|  |  | Zinc | ↑ cognitive performance (42, 43) | 0.7 to 54.4 mg/day |
|  |  | Iron | ↑ cognitive performance (42) | 1.6 to 60 mg/day |
|  |  | B12 | ↑ cognitive performance (42) | 1.04 to 1.17 µg/ day |
|  | Respiratory health | Vitamin D | ↑ incidence of sepsis (12.2%) (44)  ↓ incidence of repeat episodes of pneumonia (34%) (45) | Low vs high serum vitamin D  < 300,000 IU (single dose) |
| Teenagers (male and female) aged 12-18 years | Anxiety and depression | Vitamin D | ↓ anxiety and depression symptoms (46) | 25 µg/day to 1250 µg/week |
|  | Cognitive development | Vitamin D | ↑ cognitive function (1.22 IQ points) (38) | 60 to 1000 IU per day |
|  |  | Omega 3 fatty acids | ↑ scores on measures of cognition including learning, decision making and memory (39).  ↑ long-term memory, working memory and problem solving (40).  ↑ treatment of autism, ADHD, and psychosis (41). | ≥450 mg/day long chain omega 3 fatty acids; increase in blood levels (to >6%)  EPA (from 0 to 720 mg/day), but not DPA (from 0-1200 mg/day)  0.1 to 2.2 g/day omega-3 fatty acids |
|  |  | Zinc | ↑ cognitive performance (42). | 1.7 to 2.9 mg/day |
|  | Growth and development focusing on bone and muscle | Vitamin D | ↑ total body BMC (2.6%), lumbar spine BMC (1.7%) (in subjects with low serum vitamin D) (33, 34). | 132 IU/day to 14000 IU/week for at least 3 months |
|  |  | Calcium | ↑ femoral neck BMD (increased 0.02 g/cm2) and hip BMD (0.03 g/cm^2^) (35)  ↑ total body BMC (0.14 g), upper limb BMD (increase of 0.14 g/cm^2^) (36, 37). | Increase in calcium intake by 460 mg/day  300-1200 mg/day |
|  | Maturation focusing puberty/sexual organ development | Fibre | ↓ early onset of menarche (17%) (47) | High vs low intake |
|  |  | MUFA | ↓ early onset of menarche (34%) (47) | High vs low intake |
| Males aged 19-60 years | Anxiety and depression | Vitamin D | ↓ depressive symptoms (48) | ≥ 50,000 IU/week |
|  |  | Omega 3 fatty acids | ↓ depressive symptoms (49) | All doses, but ≥2000 mg/day better than <2000 mg/day |
|  |  | Vitamin C | ↑ mood in people with depression (50) | 100 to 4000 mg/day |
|  |  | Folate | ↓ depressive symptoms in people with depression (51) | 0.5 to 10 mg/day folic acid; 15 mg/day L-Methylfolate |
|  |  | Zinc | ↓ depressive symptoms (in combination with antidepressant drugs) (52) | 7 to 25 mg/day; |
|  |  | Magnesium | ↑ treatment efficacy for depression (53) | 120 to 300 mg/day elemental magnesium; 250 mg/day magnesium oxide; 320 mg to 4 g/day magnesium sulphate |
|  | Cancer | Vitamin D | ↓ risk renal cell carcinoma (24%); liver cancer (22%); melanoma thickness (54-57)  ↑ overall survival (13%); ↔ progression-free survival, cancer-specific survival, or relapse (58). | High vs low blood levels  1200 to 8000 IU/day after cancer diagnosis |
|  |  | Omega 3 fatty acids | ↓ markers of inflammation  (in adults undergoing chemotherapy or radiotherapy) (59)  ↓ risk colorectal cancer (20-24%) (60) | 0.51 to 2.2 g/day EPA and 0.24 to 0.92 g/day DHA.  High vs low blood levels DPA or DHA |
|  |  | Folate | ↓ risk colorectal cancer (29%) (61, 62)  ↓ pathological changes associated with gastric pre-cancerous conditions (63) | High vs low intake  20 to 30 mg/day folic acid |
|  |  | Fibre | ↓ risk colon cancer | High vs low intake |
|  |  | Protein | ↑ risk prostate cancer (8-10%) (64-66) | ≥ 30 g/day dairy protein; dose-response per 20 g/day increase |
|  |  | Selenium | ↓ risk prostate cancer (14%) (67) | High vs low intake |
|  | CVD | Vitamin D | ↓ risk cardiovascular events (14%, CAD patients) (68)  ↔ risk of stroke, heart attack, total cardiovascular events, total cerebrovascular events, CVD mortality (69, 70)  ↓ BP; ↔ TC, TG (71) | High vs low blood levels  Any dose  1000 to 7142.9 IU/day |
|  |  | Omega 3 fatty acids | ↓ risk CVD (21-55%), CHD (15-33%); stroke (16%) (60)  ↓ risk heart attack (0-9%), CHD (7%); cardiovascular events (0-7%), cardiac death (7-8%); ↔ stroke (72-78)  ↓ TG (15%); homocysteine (1.34 mmol/L), TC ↔ LDL-C, HDL-C (75, 76, 79) | High vs low blood levels DPA, DHA, EPA; not ALA  0.5 to 5 g/day; per 1 g/day increase  0.5 to >5 g/day |
|  |  | Folate | ↓ risk stroke (20%) (72) | 0.8 mg folic acid |
|  |  | Fibre | ↓ risk CVD (23%), CHD (24%); TC, LDL-C, TG (80, 81)  ↓ risk CVD (9%), CHD (11%) (80)  ↓ SBP, DBP, TC, LDL-C; ↔TG, HDL-C (82) | High vs low intake  10 g/day increase  3 to 30 g/day |
|  |  | Plant protein | ↓ risk CVD mortality (22%) (83) | High vs low intake |
|  |  | Omega 6 fatty acids | ↓ risk CVD (7%), stroke (12%), CVD mortality (22%) (84) | High vs low intake LA |
|  | Metabolic health | Vitamin D | ↓ WHR, FBG, FBI, HOMA-IR, ↑ QUICKI; ↔ WC, BMI, %BF (71)  ↓ BMI (0.11 kg/m2), WC (0.79 cm); ↔ BW, %BF (85) | 1000 to 7142.9 IU/day  1000 IU/day to 59,000 IU periodically |
|  |  | Omega 3 fatty acids | ↓ risk T2DM (11-16%) (60) | High vs low blood markers of omega-3 intake (ALA, EPA, DPA) |
|  |  | Fibre | ↓ FBG, FBI, HOMA-IR, HbA1c (82) | 3-20 g/day |
| Females aged 19-45 years | Anxiety and depression | Vitamin D | ↓ depressive symptoms (48) | ≥ 50,000 IU/week |
|  |  | Omega 3 fatty acids | ↓ depressive symptoms (49) | All doses, but ≥2000 mg/day better than <2000 mg/day |
|  |  | Vitamin C | ↑ mood in people with depression (50) | 100 to 4000 mg/day |
|  |  | Folate | ↓ depressive symptoms in people with depression (51) | 0.5 to 10 mg/day folic acid; 15 mg/day L-Methylfolate |
|  |  | Zinc | ↓ depressive symptoms (in combination with antidepressant drugs) (52) | 7 to 25 mg/day |
|  |  | Magnesium | ↑ treatment efficacy for depression (53) | 120 to 300 mg/day elemental magnesium; 250 mg/day magnesium oxide; 320 mg to 4 g/day magnesium sulphate. |
|  | Cancer | Vitamin D | ↓ risk renal cell carcinoma (24%); liver cancer (22%); breast cancer (6%); melanoma thickness (54-57)  ↑ overall survival (13%); ↔ progression-free survival, cancer-specific survival, or relapse (58). | High vs low blood levels  1200 to 8000 IU/day after cancer diagnosis |
|  |  | Omega 3 fatty acids | ↓ markers of inflammation  (in adults undergoing chemotherapy or radiotherapy) (59)  ↓ risk colorectal cancer (20-24%) (60) | 0.51 to 2.2 g/day EPA and 0.24 to 0.92 g/day DHA.  High vs low blood levels DPA or DHA |
|  |  | Folate | ↓ risk colorectal cancer (29%) (61)  ↓pathological changes associated with gastric precancerous conditions (63) | High vs low intake  20 to 30 mg/day folic acid |
|  |  | Fibre | ↓ risk colon cancer; breast cancer (9-18%) (64, 65) | High vs low intake |
|  | CVD | Vitamin D | ↓ risk cardiovascular events (14%, CAD patients) (68)  ↔ risk of stroke, heart attack, total cardiovascular events, total cerebrovascular events, CVD mortality (69, 70)  ↓ BP; ↔ TC, TG (71) | High vs low blood levels  Any dose  1000 to 7142.9 IU/day |
|  |  | Omega 3 fatty acids | ↓ risk CVD (21-55%), CHD (15-33%); stroke (16%) (60)  ↓ risk heart attack (0-9%), CHD (7%); cardiovascular events (0-7%), cardiac death (7-8%); ↔ stroke (72-78)  ↓ TG (15%); homocysteine (1.34 mmol/L), TC; ↔ LDL-C, HDL-C (75, 76, 79) | High vs low blood levels DPA, DHA, EPA; not ALA  0.5 to 5 g/day; per 1 g/day increase  0.5 to >5 g/day |
|  |  | Folate | ↓ risk stroke (20%) (72) | 0.8 mg folic acid |
|  |  | Fibre | ↓ risk CVD (23%), CHD (24%); total cholesterol, LDL cholesterol, triglycerides (80, 81)  ↓ risk CVD (9%), CHD (11%) (80)  ↓ SBP, DBP, TC, LDL-C; ↔TG, HDL-C (82) | High vs low intake  10 g/day increase  3 to 30 g/day |
|  |  | Plant protein | ↓ risk CVD mortality (22%) (83) | High vs low intake |
|  |  | Omega 6 fatty acids | ↓ risk CVD (7%), stroke (12%), CVD mortality (22%) (84) | High vs low intake LA |
|  | Fertility and pre-pregnancy nutrition | Vitamin D | ↑ AMH levels (0.49 SMD, non-PCOS women) (86)  ↑ IVF outcomes (29-64%) (87) | 1000 IU/day; 50000 IU/week  Sufficient vs insufficient or deficient blood status |
|  |  | Omega 3 fatty acids | ↑ fertilisation rate and embryo quality (IVF) (88) | 1 to 2 g/day; high vs low intake |
|  |  | Folate | ↓ risk birth defects (63%) (89)  ↓ prevalence autism/neuro-developmental conditions (90)  ↑ risk respiratory allergic diseases in both the infant and child (91) | Any dose  0.4 to <5 mg/day folic acid  Intake < 400 µg/day folic acid |
|  | Metabolic health | Vitamin D | ↓ WHR, FBG, FBI, HOMA-IR, ↑ QUICKI; ↔ WC, BMI, %BF (71)  ↓ BMI (0.11 kg/m2), WC (0.79 cm); ↔ BW, %BF (85) | 1000 to 7142.9 IU/day  1000 IU/day to 59,000 IU periodically |
|  |  | Omega 3 fatty acids | ↓ risk T2DM (11-16%) (60) | High vs low blood markers of omega-3 intake (ALA, EPA, DPA) |
|  |  | Fibre | ↓ FBG, FBI, HOMA-IR, HbA1c (82) | 3-20 g/day |
| Pregnant or lactating females aged 19-45 years | Healthy foetal/infant growth and development. | Vitamin D | ↑ offspring length at birth (0.27-7 cm), neonatal vitamin D concentration (27.72 nmol/L); ↓ risk of vitamin D insufficiency (49%), SGA (54%), neonatal death; ↔ preterm birth (RCTs) (92-96)  ↓ risk pre-term birth (6%), small for gestational age (10%) (97, 98)  ↑ risk miscarriage (60%); ↑ live birth rate (IVF) (87, 99)  ↑ cognition and psychomotor development of the infant; ↓ risk ADHD, autism traits (100) | 800 to 7142.9 IU/day  Each 25 mmol increase in blood status  Vitamin D deficiency or insufficiency vs sufficiency  High vs low blood status |
|  |  | Omega 3 fatty acids | ↑ cognitive development in the infant or child; ↔ cognitive performance (101, 102) | 200–2200 mg/day DHA and 0–1100 mg/day EPA |
|  |  | Folate | ↓ risk birth defects (63%) (89)  ↓ prevalence autism/neuro-developmental conditions (90)  ↑ risk respiratory allergic diseases in both the infant and child (91) | Any dose  0.4 to <5 mg/day folic acid  Intake < 400 µg/day folic acid |
|  |  | Iodine | ↑ psychomotor development (children in areas of mild to moderate iodine deficiency) (103) | 300 μg/day of potassium iodide |
|  | Healthy maternal weight gain. | Vitamin D | ↓ risk preeclampsia (35-75%); ↓ blood lipids, markers of inflammation and oxidative stress (96, 104-106)  ↓ risk GDM (30-36%) (96, 106)  ↑ insulin sensitivity; ↓ FBG (10.20 mg/dL), FBI (5.02 mIU/mL), HOMA-IR (1.06 units); ↔ post-delivery (previous GDM) (107-110) | Any dose; insufficiency or deficiency  >2000 IU/day  400 to 3571.4 IU/day |
|  |  | Omega 3 fatty acids | ↓ risk preeclampsia (111) | Up to 2100 mg/day DHA; up to 3000 mg/day EPA |
|  |  | Folate | ↑ risk GDM (23-96%) (112) | High vs low blood folate; folic acid supplementation >3 months |
|  |  | Zinc | ↓ FBG, FBI, HOMA-IR (women with GDM) (113) | 8 to 30 mg/day |
|  |  | Magnesium | ↓ TC, LDL-C, BG, FBI, HOMA-IR; ↑ QUICKI (women with GDM) (114) | 250 mg/day magnesium oxide |
|  |  | Calcium | ↓ incidence preeclampsia (115) | 120 to 800 mg/day |
|  |  | Fibre | ↓ TC, LDL-C (GDM) (116) | 9.5 to 30 g/day |
|  | Maternal mental health. | Vitamin D | ↓ risk maternal depression (117, 118) | Blood vitamin D status of 90–110 nmol/l; 400 to 6000 IU/day |
|  |  | Omega 3 fatty acids | ↓ depressive symptoms (119) | 120 to 1638 mg/day DHA, up to 2200 mg/day EPA, 400 mg/day ALA |
| Peri or post-menopausal females aged >45-60 years | Anxiety and depression | Vitamin D | ↓ depressive symptoms (48) | ≥ 50,000 IU/week |
|  |  | Omega 3 fatty acids | ↓ depressive symptoms (49) | All doses, but ≥2000 mg/day better than <2000 mg/day |
|  |  | Vitamin C | ↑ mood in people with depression (50) | 100 to 4000 mg/day |
|  |  | Folate | ↓ depressive symptoms in people with depression (51) | 0.5 to 10 mg/day folic acid; 15 mg/day L-Methylfolate |
|  |  | Zinc | ↓ depressive symptoms (in combination with antidepressant drugs) (52) | 7 to 25 mg/day; |
|  |  | Magnesium | ↑ treatment efficacy for depression (53) | 120 to 300 mg/day elemental magnesium; 250 mg/day magnesium oxide; 320 mg to 4 g/day magnesium sulfate. |
|  | Bone health | Vitamin D | ↑ risk fractures, osteoporosis (120)  ↑ BMD (121) | Low vs high serum status  < 400 IU/day (combined with calcium) |
|  |  | Calcium | ↑ BMD (1.62%) (122) | ≥ 700 mg/day; optimal intake 1200 mg/day |
|  |  | Protein | ↑ lumbar BMD (123) | High vs low intake |
|  | Cancer | Vitamin D | ↓ risk renal cell carcinoma (24%); liver cancer (22%); breast cancer (6%); melanoma thickness (54-57)  ↑ overall survival (13%); ↔ progression-free survival, cancer-specific survival, or relapse (58) | High vs low blood levels  1200 to 8000 IU/day after cancer diagnosis |
|  |  | Omega 3 fatty acids | ↓ markers of inflammation  (in adults undergoing chemotherapy or radiotherapy) (59)  ↓ risk colorectal cancer (20-24%) (60) | 0.51 to 2.2 g/day EPA and 0.24 to 0.92 g/day DHA.  High vs low blood levels DPA or DHA |
|  |  | Folate | ↓ risk colorectal cancer (29%); breast cancer risk (15%) (61, 62)  ↓ pathological changes associated with gastric precancerous conditions (63) | High vs low intake  20 to 30 mg/day folic acid |
|  |  | Fibre | ↓ risk colon cancer; breast cancer (9-18%) (64, 65) | High vs low intake |
|  | CVD | Vitamin D | ↓ risk cardiovascular events (14%, CAD patients) (68)  ↔ risk of stroke, heart attack, total cardiovascular events, total cerebrovascular events, CVD mortality (69, 70)  ↓ BP; ↔ TC, TG (71) | High vs low blood levels  Any dose  1000 to 7142.9 IU/day |
|  |  | Omega 3 fatty acids | ↓ risk CVD (21-55%), CHD (15-33%); stroke (16%) (60)  ↓ risk heart attack (0-9%), CHD (7%); cardiovascular events (0-7%), cardiac death (7-8%); ↔ stroke (72-78)  ↓ TG (15%); homocysteine (1.34 mmol/L), TC; ↔ LDL-C, HDL-C (75, 76, 79) | High vs low blood levels DPA, DHA, EPA; not ALA  0.5 to 5 g/day ; per 1 g/day increase  0.5 to >5 g/day |
|  |  | Folate | ↓ risk stroke (20%) (72) | 0.8 mg folic acid |
|  |  | Fibre | ↓ risk CVD (23%), CHD (24%); total cholesterol, LDL cholesterol, triglycerides (80, 81)  ↓ risk CVD (9%), CHD (11%) (80)  ↓ SBP, DBP, TC, LDL-C; ↔ TG, HDL-C (82) | High vs low intake  10 g/day increase  3 to 30 g/day |
|  |  | Plant protein | ↓ risk CVD mortality (22%) (83) | High vs low intake |
|  |  | Omega 6 fatty acids | ↓ risk CVD (7%), stroke (12%), CVD mortality (22%) (84) | High vs low intake LA |
|  | Metabolic health | Vitamin D | ↓ WHR, FBG, FBI, HOMA-IR, ↑ QUICKI; ↔ WC, BMI, %BF (71)  ↓ BMI (0.11 kg/m2), WC (0.79 cm); ↔ BW, %BF (85) | 1000 to 7142.9 IU/day  1000 IU/day to 59,000 IU periodically |
|  |  | Omega 3 fatty acids | ↓ risk T2DM (11-16%) (60) | High vs low blood markers of omega-3 intake (ALA, EPA, DPA) |
|  |  | Fibre | ↓ FBG, FBI, HOMA-IR, HbA1c (82) | 3-20 g/day |
| Older adults (males and females) aged >60 years | Cognitive function / decline | Vitamin D | ↑ risk post-operative delirium and cognitive dysfunction (54%) (124) | Vitamin D deficiency |
|  |  | Omega 3 fatty acids | ↑ cognitive function, attention, orientation, perception, verbal functions, language skills (125-127) | 180 to 2000 mg/day DHA; 40 to 1080 mg per day EPA |
|  | CVD | Vitamin D | ↓ risk cardiovascular events (14%, CAD patients) (68)  ↔ risk of stroke, heart attack, total cardiovascular events, total cerebrovascular events, CVD mortality (69, 70)  ↓ BP; ↔ TC, TG (71) | High vs low blood levels  Any dose  1000 to 7142.9 IU/day |
|  |  | Omega 3 fatty acids | ↓ risk CVD (21-55%), CHD (15-33%); stroke (16%) (60)  ↓ risk heart attack (0-9%), CHD (7%); cardiovascular events (0-7%), cardiac death (7-8%); ↔ stroke (72-78)  ↓ TG (15%); homocysteine (1.34 mmol/L),TC; ↔ LDL-C, HDL-C (75, 76, 79) | High vs low blood levels DPA, DHA, EPA; not ALA  0.5 to 5 g/day ; per 1 g/day increase  0.5 to >5 g/day |
|  |  | Folate | ↓ risk stroke (20%) (72) | 0.8 mg folic acid |
|  |  | Fibre | ↓ risk CVD (23%), CHD (24%); total cholesterol, LDL cholesterol, triglycerides (80, 81)  ↓ risk CVD (9%), CHD (11%) (80)  ↓SBP, DBP, TC, LDL-C; ↔TG, HDL-C (82) | High vs low intake  10 g/day increase  3 to 30 g/day |
|  |  | Plant protein | ↓ risk CVD mortality (22%) (83) | High vs low intake |
|  |  | Omega 6 fatty acids | ↓ risk CVD (7%), stroke (12%), CVD mortality (22%) (84) | High vs low intake LA |
|  | Physical independence (i.e., muscle and bone maintenance, falls risk) | Vitamin D | ↑ strength, balance (128)  ↑ handgrip strength, lower limb strength, physical performance (129-131)  ↓ incidence falls (132)  ↓ physical performance scores (133)  ↓ risk hip fracture (52%) (134)  ↓ risk falls (12-23%) (135) | 800 to 1000 IU  >1000 IU/day; 500-1600 IU/day; 2500-5000 IU/week (with protein or exercise)  >700 IU/day  1000 to 50,000 IU/day  High vs low serum status  400-60 000 IU/day (with insufficient serum status); 700-1000 IU (combined with calcium |
|  |  | Omega 3 fatty acids | ↑ lower body strength, timed-up-and-go performance, and 30-second sit-to-stand performance, lean body mass; ↔ walking performance, upper body strength, handgrip strength (136-138) | 0.23 to 5 g/day EPA + DHA; 1.35 to 14 g/day ALA; 1.3 to 5 g/day total fatty acids |
|  |  | Protein/ EAA/BCAA/ leucine | ↑ protein synthesis, LBM, muscle strength, muscle performance, aerobic capacity, hand grip strength; ↓ risk sarcopenia, falls; ↓ %BF (139-144)  ↓ risk frailty (145)  ↓ risk sarcopenia; ↑ muscle strength, performance (146-148) | 1.2 to 1.8 g/kg bw/day protein; 8.5 to 100 g/day protein; 2.5 to 15 g/day EAA or BCAA  High vs low protein intake  0.9-7.5 g/day leucine |
|  | Metabolic health | Vitamin D | ↓ WHR, FBG, FBI, HOMA-IR, ↑ QUICKI; ↔ WC, BMI, %BF (71)  ↓ BMI (0.11 kg/m2), WC (0.79 cm); ↔ BW, %BF (85) | 1000 to 7142.9 IU/day  1000 IU/day to 59,000 IU periodically |
|  |  | Omega 3 fatty acids | ↓ risk T2DM (11-16%) (60) | High vs low blood markers of omega-3 intake (ALA, EPA, DPA) |
|  |  | Fibre | ↓ FBG, FBI, HOMA-IR, HbA1c (82) | 3-20 g/day |

ADHD, Attention Deficit Hyperactivity Disorder; ALA, alpha linolenic acid; AMH, anti-Müllerian hormone; BMC, bone mineral content; BMD, bone mineral density; BMI, body mass index; BP, blood pressure; BW, bodyweight; CAD, coronary artery disease; CHD, coronary heart disease; CVD, cardiovascular disease; DHA, docosahexanoic acid; DPA, docosopentanoic acid; EPA, eicosapentanoic acid; FBG, fasting blood glucose; FBI, fasting blood insulin; GDM, gestational diabetes mellitus; HbA1c, glycosylated haemoglobin; HDL-C, high density lipoprotein cholesterol; HOMA-IR, Homeostasis Model Assessment for Insulin Resistance; IQ, Intelligence Quotient; IU, international units; IVF, *in vitro* fertilisation; LA, linoleic acid; LBM, lean body mass; LDL-C, low density lipoprotein cholesterol; PCOS, polycystic ovarian syndrome; QUICKI, Quantitative Insulin Sensitivity Check Index; RCT, randomised controlled trial; SGA, small for gestational age; SMD, standardised mean difference; T2DM, type 2 diabetes mellitus; TC, total cholesterol; TG, triglycerides; WC, waist circumference; WHR, waist to hip ratio; %BF, percentage body fat.

1. Parnell P, Scragg R, Wilson N, Schaaf D, Fitzgerald E. NZ Food NZ Children. Key results of the 2002 National Children’s Nutrition Survey. Wellington, NZ: Ministry of Health; 2003.

2. Australian Health Survey: Usual Nutrient Intakes: Australian Bureau of Statistics; 2015 [Available from: <https://www.abs.gov.au/statistics/health/health-conditions-and-risks/australian-health-survey-usual-nutrient-intakes/2011-12>.

3. Probst Y, Guan V, Neale E. Development of a Choline Database to Estimate Australian Population Intakes. Nutrients. 2019;11(4).

4. Cressey P, Gaw S, Love J. Estimated dietary fluoride intake for New Zealanders. Institute of Environmental Science & Research Limited; 2009.

5. Mackay S, Parnell W, Heath A-L, Brown R, Wilson N, Gray A, et al. A Focus on Nutrition. Key Findings of the 2008/09 New Zealand Adult Nutrition Survey. Wellington, NZ: University of Otago

and Ministry of Health; 2011.

6. Fenech M, Baghurst P, Luderer W, Turner J, Record S, Ceppi M, Bonassi S. Low intake of calcium, folate, nicotinic acid, vitamin E, retinol, beta-carotene and high intake of pantothenic acid, biotin and riboflavin are significantly associated with increased genome instability--results from a dietary intake and micronucleus index survey in South Australia. Carcinogenesis. 2005;26(5):991-9.

7. Slater K, Rollo ME, Szewczyk Z, Ashton L, Schumacher T, Collins C. Do the Dietary Intakes of Pregnant Women Attending Public Hospital Antenatal Clinics Align with Australian Guide to Healthy Eating Recommendations? Nutrients. 2020;12(8):2438.

8. Watson PE, McDonald BW. Major influences on nutrient intake in pregnant New Zealand women. Matern Child Health J. 2009;13(5):695-706.

9. Watson PE, McDonald BW. The association of maternal diet and dietary supplement intake in pregnant New Zealand women with infant birthweight. Eur J Clin Nutr. 2010;64(2):184-93.

10. Butts CA, Hedderley DI, Herath TD, Paturi G, Glyn-Jones S, Wiens F, et al. Human Milk Composition and Dietary Intakes of Breastfeeding Women of Different Ethnicity from the Manawatu-Wanganui Region of New Zealand. Nutrients. 2018;10(9).

11. Ekeroma AJ, Camargo CA, Jr., Scragg R, Wall C, Stewart A, Mitchell E, et al. Predictors of vitamin D status in pregnant women in New Zealand. N Z Med J. 2015;128(1422):24-34.

12. Nutrient Reference Values for Australia and New Zealand Including Recommended Dietary Intakes. Canberra: National Health and Medical Resarch Council; 2005.

13. Hudson JL, Baum JI, Diaz EC, Børsheim E. Dietary Protein Requirements in Children: Methods for Consideration. Nutrients. 2021;13(5).

14. Abrams SA, Chen Z, Hawthorne KM. Magnesium Metabolism in 4‐Year‐Old to 8‐Year‐Old Children. Journal of Bone and Mineral Research. 2014;29(1):118-22.

15. Nielsen FH. The Problematic Use of Dietary Reference Intakes to Assess Magnesium Status and Clinical Importance. Biol Trace Elem Res. 2019;188(1):52-9.

16. Krebs NF. Bioavailability of dietary supplements and impact of physiologic state: infants, children and adolescents. The Journal of nutrition. 2001;131(4):1351S-4S.

17. Armah SM. Fractional zinc absorption for men, women, and adolescents is overestimated in the current dietary reference intakes. The Journal of nutrition. 2016;146(6):1276-80.

18. Elango R, Humayun MA, Ball RO, Pencharz PB. Evidence that protein requirements have been significantly underestimated. Curr Opin Clin Nutr Metab Care. 2010;13(1):52-7.

19. Humayun MA, Elango R, Ball RO, Pencharz PB. Reevaluation of the protein requirement in young men with the indicator amino acid oxidation technique. Am J Clin Nutr. 2007;86(4):995-1002.

20. Levine M, Conry-Cantilena C, Wang Y, Welch RW, Washko PW, Dhariwal KR, et al. Vitamin C pharmacokinetics in healthy volunteers: evidence for a recommended dietary allowance. Proceedings of the National Academy of Sciences. 1996;93(8):3704-9.

21. Nutrient Reference Values for Australia and New Zealand: National Health and Medical Research Council; [Available from: <https://www.nrv.gov.au/>.

22. Lim KH, Riddell LJ, Nowson CA, Booth AO, Szymlek-Gay EA. Iron and zinc nutrition in the economically-developed world: a review. Nutrients. 2013;5(8):3184-211.

23. Fayet-Moore F, Petocz P, Samman S. Micronutrient Status in Female University Students: Iron, Zinc, Copper, Selenium, Vitamin B12 and Folate. Nutrients. 2014;6(11):5103-16.

24. Hujoel PP, Hujoel MLA. Vitamin C and scar strength: analysis of a historical trial and implications for collagen-related pathologies. The American Journal of Clinical Nutrition. 2021;115(1):8-17.

25. Berti C, Biesalski H, Gärtner R, Lapillonne A, Pietrzik K, Poston L, et al. Micronutrients in pregnancy: current knowledge and unresolved questions. Clinical nutrition. 2011;30(6):689-701.

26. Berti C, Decsi T, Dykes F, Hermoso M, Koletzko B, Massari M, et al. Critical issues in setting micronutrient recommendations for pregnant women: an insight. Maternal & child nutrition. 2010;6:5-22.

27. Fung EB, Ritchie LD, Woodhouse LR, Roehl R, King JC. Zinc absorption in women during pregnancy and lactation: a longitudinal study. The American Journal of Clinical Nutrition. 1997;66(1):80-8.

28. Montgomery SC, Streit SM, Beebe ML, Maxwell IV PJ. Micronutrient needs of the elderly. Nutrition in clinical practice. 2014;29(4):435-44.

29. Baum JI, Kim I-Y, Wolfe RR. Protein Consumption and the Elderly: What Is the Optimal Level of Intake? Nutrients. 2016;8(6):359.

30. Dorrington N, Fallaize R, Hobbs DA, Weech M, Lovegrove JA. A review of nutritional requirements of adults aged≥ 65 years in the UK. The Journal of nutrition. 2020;150(9):2245-56.

31. ter Borg S, Verlaan S, Hemsworth J, Mijnarends DM, Schols JM, Luiking YC, de Groot LC. Micronutrient intakes and potential inadequacies of community-dwelling older adults: a systematic review. British Journal of Nutrition. 2015;113(8):1195-206.

32. Chernoff R. Micronutrient requirements in older women. The American journal of clinical nutrition. 2005;81(5):1240S-5S.

33. Winzenberg T, Powell S, Shaw KA, Jones G. Effects of vitamin D supplementation on bone density in healthy children: systematic review and meta-analysis. Bmj. 2011;342:c7254.

34. Winzenberg TM, Powell S, Shaw KA, Jones G. Vitamin D supplementation for improving bone mineral density in children. Cochrane Database Syst Rev. 2010(10):Cd006944.

35. Cormick G, Betran AP, Romero IB, Cormick MS, Belizán JM, Bardach A, Ciapponi A. Effect of Calcium Fortified Foods on Health Outcomes: A Systematic Review and Meta-Analysis. Nutrients. 2021;13(2).

36. Winzenberg TM, Shaw K, Fryer J, Jones G. Calcium supplementation for improving bone mineral density in children. Cochrane Database Syst Rev. 2006(2):Cd005119.

37. Winzenberg T, Shaw K, Fryer J, Jones G. Effects of calcium supplementation on bone density in healthy children: meta-analysis of randomised controlled trials. Bmj. 2006;333(7572):775.

38. Al Khalifah R, Alsheikh R, Alnasser Y, Alsheikh R, Alhelali N, Naji A, Al Backer N. The impact of vitamin D food fortification and health outcomes in children: a systematic review and meta-regression. Syst Rev. 2020;9(1):144.

39. van der Wurff ISM, Meyer BJ, de Groot RHM. Effect of Omega-3 Long Chain Polyunsaturated Fatty Acids (n-3 LCPUFA) Supplementation on Cognition in Children and Adolescents: A Systematic Literature Review with a Focus on n-3 LCPUFA Blood Values and Dose of DHA and EPA. Nutrients. 2020;12(10).

40. Emery S, Häberling I, Berger G, Walitza S, Schmeck K, Albert T, et al. Omega-3 and its domain-specific effects on cognitive test performance in youths: A meta-analysis. Neurosci Biobehav Rev. 2020;112:420-36.

41. Agostoni C, Nobile M, Ciappolino V, Delvecchio G, Tesei A, Turolo S, et al. The Role of Omega-3 Fatty Acids in Developmental Psychopathology: A Systematic Review on Early Psychosis, Autism, and ADHD. Int J Mol Sci. 2017;18(12).

42. Meli AM, Ali A, Mhd Jalil AM, Mohd Yusof H, Tan MMC. Effects of Physical Activity and Micronutrients on Cognitive Performance in Children Aged 6 to 11 Years: A Systematic Review and Meta-Analysis of Randomized Controlled Trials. Medicina (Kaunas). 2021;58(1).

43. Tsang BL, Holsted E, McDonald CM, Brown KH, Black R, Mbuya MNN, et al. Effects of Foods Fortified with Zinc, Alone or Cofortified with Multiple Micronutrients, on Health and Functional Outcomes: A Systematic Review and Meta-Analysis. Adv Nutr. 2021;12(5):1821-37.

44. Yu W, Ying Q, Zhu W, Huang L, Hou Q. Vitamin D status was associated with sepsis in critically ill children: A PRISMA compliant systematic review and meta-analysis. Medicine (Baltimore). 2021;100(2):e23827.

45. Yang C, Lu Y, Wan M, Xu D, Yang X, Yang L, et al. Efficacy of High-Dose Vitamin D Supplementation as an Adjuvant Treatment on Pneumonia: Systematic Review and a Meta-Analysis of Randomized Controlled Studies. Nutrition in Clinical Practice. 2021;36(2):368-84.

46. Głąbska D, Kołota A, Lachowicz K, Skolmowska D, Stachoń M, Guzek D. The Influence of Vitamin D Intake and Status on Mental Health in Children: A Systematic Review. Nutrients. 2021;13(3).

47. Nguyen NTK, Fan HY, Tsai MC, Tung TH, Huynh QTV, Huang SY, Chen YC. Nutrient Intake through Childhood and Early Menarche Onset in Girls: Systematic Review and Meta-Analysis. Nutrients. 2020;12(9).

48. Jamilian H, Amirani E, Milajerdi A, Kolahdooz F, Mirzaei H, Zaroudi M, et al. The effects of vitamin D supplementation on mental health, and biomarkers of inflammation and oxidative stress in patients with psychiatric disorders: A systematic review and meta-analysis of randomized controlled trials. Prog Neuropsychopharmacol Biol Psychiatry. 2019;94:109651.

49. Luo XD, Feng JS, Yang Z, Huang QT, Lin JD, Yang B, et al. High-dose omega-3 polyunsaturated fatty acid supplementation might be more superior than low-dose for major depressive disorder in early therapy period: a network meta-analysis. BMC Psychiatry. 2020;20(1):248.

50. Yosaee S, Keshtkaran Z, Abdollahi S, Shidfar F, Sarris J, Soltani S. The effect of vitamin C supplementation on mood status in adults: a systematic review and meta-analysis of randomized controlled clinical trials. Gen Hosp Psychiatry. 2021;71:36-42.

51. Altaf R, Gonzalez I, Rubino K, Nemec EC, 2nd. Folate as adjunct therapy to SSRI/SNRI for major depressive disorder: Systematic review & meta-analysis. Complement Ther Med. 2021;61:102770.

52. da Silva LEM, de Santana MLP, Costa PRF, Pereira EM, Nepomuceno CMM, Queiroz VAO, et al. Zinc supplementation combined with antidepressant drugs for treatment of patients with depression: a systematic review and meta-analysis. Nutr Rev. 2021;79(1):1-12.

53. Botturi A, Ciappolino V, Delvecchio G, Boscutti A, Viscardi B, Brambilla P. The Role and the Effect of Magnesium in Mental Disorders: A Systematic Review. Nutrients. 2020;12(6).

54. Guo XF, Zhao T, Han JM, Li S, Li D. Vitamin D and liver cancer risk: A meta-analysis of prospective studies. Asia Pac J Clin Nutr. 2020;29(1):175-82.

55. Song D, Deng Y, Liu K, Zhou L, Li N, Zheng Y, et al. Vitamin D intake, blood vitamin D levels, and the risk of breast cancer: a dose-response meta-analysis of observational studies. Aging (Albany NY). 2019;11(24):12708-32.

56. Song Y, Lu H, Cheng Y. To identify the association between dietary vitamin D intake and serum levels and risk or prognostic factors for melanoma-systematic review and meta-analysis. BMJ Open. 2022;12(8):e052442.

57. Wu J, Yang N, Yuan M. Dietary and circulating vitamin D and risk of renal cell carcinoma: a meta-analysis of observational studies. Int Braz J Urol. 2021;47(4):733-44.

58. Chen QY, Kim S, Lee B, Jeong G, Lee DH, Keum N, et al. Post-Diagnosis Vitamin D Supplement Use and Survival among Cancer Patients: A Meta-Analysis. Nutrients. 2022;14(16).

59. Tao X, Zhou Q, Rao Z. Efficacy of ω-3 Polyunsaturated Fatty Acids in Patients with Lung Cancer Undergoing Radiotherapy and Chemotherapy: A Meta-Analysis. Int J Clin Pract. 2022;2022:6564466.

60. Jiang H, Wang L, Wang D, Yan N, Li C, Wu M, et al. Omega-3 polyunsaturated fatty acid biomarkers and risk of type 2 diabetes, cardiovascular disease, cancer, and mortality. Clin Nutr. 2022;41(8):1798-807.

61. Moazzen S, Dolatkhah R, Tabrizi JS, Shaarbafi J, Alizadeh BZ, de Bock GH, Dastgiri S. Folic acid intake and folate status and colorectal cancer risk: A systematic review and meta-analysis. Clin Nutr. 2018;37(6 Pt A):1926-34.

62. Ren X, Xu P, Zhang D, Liu K, Song D, Zheng Y, et al. Association of folate intake and plasma folate level with the risk of breast cancer: a dose-response meta-analysis of observational studies. Aging (Albany NY). 2020;12(21):21355-75.

63. Lei J, Ren F, Li W, Guo X, Liu Q, Gao H, et al. Use of folic acid supplementation to halt and even reverse the progression of gastric precancerous conditions: a meta-analysis. BMC Gastroenterol. 2022;22(1):370.

64. Ma Y, Hu M, Zhou L, Ling S, Li Y, Kong B, Huang P. Dietary fiber intake and risks of proximal and distal colon cancers: A meta-analysis. Medicine (Baltimore). 2018;97(36):e11678.

65. Farvid MS, Spence ND, Holmes MD, Barnett JB. Fiber consumption and breast cancer incidence: A systematic review and meta-analysis of prospective studies. Cancer. 2020;126(13):3061-75.

66. Alzahrani MA, Shakil Ahmad M, Alkhamees M, Aljuhayman A, Binsaleh S, Tiwari R, Almannie R. Dietary protein intake and prostate cancer risk in adults: A systematic review and dose-response meta-analysis of prospective cohort studies. Complement Ther Med. 2022;70:102851.

67. Sayehmiri K, Azami M, Mohammadi Y, Soleymani A, Tardeh Z. The association between Selenium and Prostate Cancer: a Systematic Review and Meta-Analysis. Asian Pac J Cancer Prev. 2018;19(6):1431-7.

68. Zhang H, Wang P, Jie Y, Sun Y, Wang X, Fan Y. Predictive value of 25-hydroxyvitamin D level in patients with coronary artery disease: A meta-analysis. Front Nutr. 2022;9:984487.

69. Fu J, Sun J, Zhang C. Vitamin D supplementation and risk of stroke: A meta-analysis of randomized controlled trials. Front Neurol. 2022;13:970111.

70. Pei YY, Zhang Y, Peng XC, Liu ZR, Xu P, Fang F. Association of Vitamin D Supplementation with Cardiovascular Events: A Systematic Review and Meta-Analysis. Nutrients. 2022;14(15).

71. Qi KJ, Zhao ZT, Zhang W, Yang F. The impacts of vitamin D supplementation in adults with metabolic syndrome: A systematic review and meta-analysis of randomized controlled trials. Front Pharmacol. 2022;13:1033026.

72. Khan SU, Khan MU, Riaz H, Valavoor S, Zhao D, Vaughan L, et al. Effects of Nutritional Supplements and Dietary Interventions on Cardiovascular Outcomes: An Umbrella Review and Evidence Map. Ann Intern Med. 2019;171(3):190-8.

73. Yu F, Qi S, Ji Y, Wang X, Fang S, Cao R. Effects of omega-3 fatty acid on major cardiovascular outcomes: A systematic review and meta-analysis. Medicine (Baltimore). 2022;101(30):e29556.

74. Bernasconi AA, Wiest MM, Lavie CJ, Milani RV, Laukkanen JA. Effect of Omega-3 Dosage on Cardiovascular Outcomes: An Updated Meta-Analysis and Meta-Regression of Interventional Trials. Mayo Clin Proc. 2021;96(2):304-13.

75. Abdelhamid AS, Brown TJ, Brainard JS, Biswas P, Thorpe GC, Moore HJ, et al. Omega-3 fatty acids for the primary and secondary prevention of cardiovascular disease. Cochrane Database Syst Rev. 2020;3(3):Cd003177.

76. Xu Q, Du L, Gu H, Ji M, Zhan L. The effect of omega-3 polyunsaturated fatty acids on stroke treatment and prevention: a systematic review and meta-analysis. Nutr Hosp. 2022;39(4):924-35.

77. Marston NA, Giugliano RP, Im K, Silverman MG, O'Donoghue ML, Wiviott SD, et al. Association Between Triglyceride Lowering and Reduction of Cardiovascular Risk Across Multiple Lipid-Lowering Therapeutic Classes: A Systematic Review and Meta-Regression Analysis of Randomized Controlled Trials. Circulation. 2019;140(16):1308-17.

78. Yokoyama Y, Kuno T, Morita SX, Slipczuk L, Takagi H, Briasoulis A, et al. Eicosapentaenoic Acid for Cardiovascular Events Reduction- Systematic Review and Network Meta-Analysis of Randomized Controlled Trials. J Cardiol. 2022;80(5):416-22.

79. Sohouli MH, Roshan MM, Olusola OF, Fatahi S, Omidi HR, Sharifi P, et al. Impact of Omega-3 supplementation on homocysteine levels in humans: A systematic review and meta-regression analysis of randomized controlled trials. Nutr Metab Cardiovasc Dis. 2022;32(9):2013-25.

80. Kim Y, Je Y. Dietary fibre intake and mortality from cardiovascular disease and all cancers: A meta-analysis of prospective cohort studies. Arch Cardiovasc Dis. 2016;109(1):39-54.

81. Reynolds AN, Akerman AP, Mann J. Dietary fibre and whole grains in diabetes management: Systematic review and meta-analyses. PLoS Med. 2020;17(3):e1003053.

82. Fu L, Zhang G, Qian S, Zhang Q, Tan M. Associations between dietary fiber intake and cardiovascular risk factors: An umbrella review of meta-analyses of randomized controlled trials. Front Nutr. 2022;9:972399.

83. Naghshi S, Sadeghi O, Willett WC, Esmaillzadeh A. Dietary intake of total, animal, and plant proteins and risk of all cause, cardiovascular, and cancer mortality: systematic review and dose-response meta-analysis of prospective cohort studies. Bmj. 2020;370:m2412.

84. Marklund M, Wu JHY, Imamura F, Del Gobbo LC, Fretts A, de Goede J, et al. Biomarkers of Dietary Omega-6 Fatty Acids and Incident Cardiovascular Disease and Mortality. Circulation. 2019;139(21):2422-36.

85. Musazadeh V, Zarezadeh M, Ghalichi F, Kalajahi FH, Ghoreishi Z. Vitamin D supplementation positively affects anthropometric indices: Evidence obtained from an umbrella meta-analysis. Front Nutr. 2022;9:980749.

86. Moridi I, Chen A, Tal O, Tal R. The Association between Vitamin D and Anti-Müllerian Hormone: A Systematic Review and Meta-Analysis. Nutrients. 2020;12(6).

87. Iliuta F, Pijoan JI, Lainz L, Exposito A, Matorras R. Women's vitamin D levels and IVF results: a systematic review of the literature and meta-analysis, considering three categories of vitamin status (replete, insufficient and deficient). Hum Fertil (Camb). 2022;25(2):228-46.

88. Abodi M, De Cosmi V, Parazzini F, Agostoni C. Omega-3 fatty acids dietary intake for oocyte quality in women undergoing assisted reproductive techniques: A systematic review. Eur J Obstet Gynecol Reprod Biol. 2022;275:97-105.

89. Partap U, Chowdhury R, Taneja S, Bhandari N, De Costa A, Bahl R, Fawzi W. Preconception and periconception interventions to prevent low birth weight, small for gestational age and preterm birth: a systematic review and meta-analysis. BMJ Glob Health. 2022;7(8).

90. Gao Y, Sheng C, Xie RH, Sun W, Asztalos E, Moddemann D, et al. New Perspective on Impact of Folic Acid Supplementation during Pregnancy on Neurodevelopment/Autism in the Offspring Children - A Systematic Review. PLoS One. 2016;11(11):e0165626.

91. Chen Z, Xing Y, Yu X, Dou Y, Ma D. Effect of Folic Acid Intake on Infant and Child Allergic Diseases: Systematic Review and Meta-Analysis. Front Pediatr. 2020;8:615406.

92. Liu Y, Ding C, Xu R, Wang K, Zhang D, Pang W, et al. Effects of vitamin D supplementation during pregnancy on offspring health at birth: A meta-analysis of randomized controlled trails. Clin Nutr. 2022;41(7):1532-40.

93. Bi WG, Nuyt AM, Weiler H, Leduc L, Santamaria C, Wei SQ. Association Between Vitamin D Supplementation During Pregnancy and Offspring Growth, Morbidity, and Mortality: A Systematic Review and Meta-analysis. JAMA Pediatr. 2018;172(7):635-45.

94. Luo T, Lin Y, Lu J, Lian X, Guo Y, Han L, Guo Y. Effects of vitamin D supplementation during pregnancy on bone health and offspring growth: A systematic review and meta-analysis of randomized controlled trials. PLoS One. 2022b;17(10):e0276016.

95. Tareke AA, Alem A, Debebe W, Bayileyegn NS, Abebe MS, Abdu H, Zerfu TA. Maternal vitamin D and growth of under-five children: a systematic review and meta-analysis of observational and interventional studies. Glob Health Action. 2022;15(1):2102712.

96. Irwinda R, Hiksas R, Lokeswara AW, Wibowo N. Vitamin D supplementation higher than 2000 IU/day compared to lower dose on maternal-fetal outcome: Systematic review and meta-analysis. Womens Health (Lond). 2022;18:17455057221111066.

97. Fang K, He Y, Mu M, Liu K. Maternal vitamin D deficiency during pregnancy and low birth weight: a systematic review and meta-analysis. J Matern Fetal Neonatal Med. 2021;34(7):1167-73.

98. Zhao R, Zhou L, Wang S, Yin H, Yang X, Hao L. Effect of maternal vitamin D status on risk of adverse birth outcomes: a systematic review and dose-response meta-analysis of observational studies. Eur J Nutr. 2022;61(6):2881-907.

99. Tamblyn JA, Pilarski NSP, Markland AD, Marson EJ, Devall A, Hewison M, et al. Vitamin D and miscarriage: a systematic review and meta-analysis. Fertil Steril. 2022;118(1):111-22.

100. García-Serna AM, Morales E. Neurodevelopmental effects of prenatal vitamin D in humans: systematic review and meta-analysis. Mol Psychiatry. 2020;25(10):2468-81.

101. Nevins JEH, Donovan SM, Snetselaar L, Dewey KG, Novotny R, Stang J, et al. Omega-3 Fatty Acid Dietary Supplements Consumed During Pregnancy and Lactation and Child Neurodevelopment: A Systematic Review. J Nutr. 2021;151(11):3483-94.

102. Lehner A, Staub K, Aldakak L, Eppenberger P, Rühli F, Martin RD, Bender N. Impact of omega-3 fatty acid DHA and EPA supplementation in pregnant or breast-feeding women on cognitive performance of children: systematic review and meta-analysis. Nutr Rev. 2021;79(5):585-98.

103. Machamba AAL, Azevedo FM, Fracalossi KO, do CCFS. Effect of iodine supplementation in pregnancy on neurocognitive development on offspring in iodine deficiency areas: a systematic review. Arch Endocrinol Metab. 2021;65(3):352-67.

104. Fogacci S, Fogacci F, Banach M, Michos ED, Hernandez AV, Lip GYH, et al. Vitamin D supplementation and incident preeclampsia: A systematic review and meta-analysis of randomized clinical trials. Clin Nutr. 2020;39(6):1742-52.

105. Hu KL, Zhang CX, Chen P, Zhang D, Hunt S. Vitamin D Levels in Early and Middle Pregnancy and Preeclampsia, a Systematic Review and Meta-Analysis. Nutrients. 2022;14(5).

106. Chan KY, Wong MMH, Pang SSH, Lo KKH. Dietary supplementation for gestational diabetes prevention and management: a meta-analysis of randomized controlled trials. Arch Gynecol Obstet. 2021;303(6):1381-91.

107. Gallo S, McDermid JM, Al-Nimr RI, Hakeem R, Moreschi JM, Pari-Keener M, et al. Vitamin D Supplementation during Pregnancy: An Evidence Analysis Center Systematic Review and Meta-Analysis. J Acad Nutr Diet. 2020;120(5):898-924.e4.

108. Wang M, Chen Z, Hu Y, Wang Y, Wu Y, Lian F, et al. The effects of vitamin D supplementation on glycemic control and maternal-neonatal outcomes in women with established gestational diabetes mellitus: A systematic review and meta-analysis. Clinical Nutrition. 2021;40(5):3148-57.

109. Lo ACQ, Lo CCW. The effect of vitamin D supplementation on glycemic control/glucose metabolism and maternal-neonatal outcomes in women with established gestational diabetes mellitus: An updated meta-analysis. Clin Nutr. 2022;41(10):2420-3.

110. Kron-Rodrigues MR, Rudge MVC, Lima SAM. Supplementation of Vitamin D in the Postdelivery Period of Women with Previous Gestational Diabetes Mellitus: Systematic Review and Meta-Analysis of Randomized Trials. Rev Bras Ginecol Obstet. 2021;43(9):699-709.

111. Bakouei F, Delavar MA, Mashayekh-Amiri S, Esmailzadeh S, Taheri Z. Efficacy of n-3 fatty acids supplementation on the prevention of pregnancy induced-hypertension or preeclampsia: A systematic review and meta-analysis. Taiwan J Obstet Gynecol. 2020;59(1):8-15.

112. Li N, Jiang J, Guo L. Effects of maternal folate and vitamin B12 on gestational diabetes mellitus: a dose-response meta-analysis of observational studies. Eur J Clin Nutr. 2022;76(11):1502-12.

113. Li X, Zhao J. The influence of zinc supplementation on metabolic status in gestational diabetes: a meta-analysis of randomized controlled studies. J Matern Fetal Neonatal Med. 2021;34(13):2140-5.

114. Tan X, Huang Y. Magnesium supplementation for glycemic status in women with gestational diabetes: a systematic review and meta-analysis. Gynecol Endocrinol. 2022;38(3):202-6.

115. Woo Kinshella ML, Sarr C, Sandhu A, Bone JN, Vidler M, Moore SE, et al. Calcium for pre-eclampsia prevention: A systematic review and network meta-analysis to guide personalised antenatal care. Bjog. 2022;129(11):1833-43.

116. Sun J, Wang J, Ma W, Miao M, Sun G. Effects of Additional Dietary Fiber Supplements on Pregnant Women with Gestational Diabetes: A Systematic Review and Meta-Analysis of Randomized Controlled Studies. Nutrients. 2022;14(21).

117. Tan Q, Liu S, Chen D. Poor vitamin D status and the risk of maternal depression: a dose-response meta-analysis of observational studies. Public Health Nutr. 2021;24(8):2161-70.

118. Ribamar A, Almeida B, Soares A, Peniche B, Jesus P, Cruz SPD, Ramalho A. Relationship between vitamin D deficiency and both gestational and postpartum depression. Nutr Hosp. 2020;37(6):1238-45.

119. Mocking RJT, Steijn K, Roos C, Assies J, Bergink V, Ruhé HG, Schene AH. Omega-3 Fatty Acid Supplementation for Perinatal Depression: A Meta-Analysis. J Clin Psychiatry. 2020;81(5).

120. Gaugris S, Heaney RP, Boonen S, Kurth H, Bentkover JD, Sen SS. Vitamin D inadequacy among post-menopausal women: a systematic review. Qjm. 2005;98(9):667-76.

121. Liu C, Kuang X, Li K, Guo X, Deng Q, Li D. Effects of combined calcium and vitamin D supplementation on osteoporosis in postmenopausal women: a systematic review and meta-analysis of randomized controlled trials. Food Funct. 2020;11(12):10817-27.

122. Wu J, Xu L, Lv Y, Dong L, Zheng Q, Li L. Quantitative analysis of efficacy and associated factors of calcium intake on bone mineral density in postmenopausal women. Osteoporos Int. 2017;28(6):2003-10.

123. Shams-White MM, Chung M, Du M, Fu Z, Insogna KL, Karlsen MC, et al. Dietary protein and bone health: a systematic review and meta-analysis from the National Osteoporosis Foundation. The American Journal of Clinical Nutrition. 2017;105(6):1528-43.

124. Hung KC, Wang LK, Lin YT, Yu CH, Chang CY, Sun CK, Chen JY. Association of preoperative vitamin D deficiency with the risk of postoperative delirium and cognitive dysfunction: A meta-analysis. J Clin Anesth. 2022;79:110681.

125. Martí Del Moral A, Fortique F. Omega-3 fatty acids and cognitive decline: a systematic review. Nutr Hosp. 2019;36(4):939-49.

126. Zhang X, Han H, Ge X, Liu L, Wang T, Yu H. Effect of n-3 long-chain polyunsaturated fatty acids on mild cognitive impairment: a meta-analysis of randomized clinical trials. Eur J Clin Nutr. 2020;74(4):548-54.

127. McGrattan A, van Aller C, Narytnyk A, Reidpath D, Keage H, Mohan D, et al. Nutritional interventions for the prevention of cognitive impairment and dementia in developing economies in East-Asia: a systematic review and meta-analysis. Crit Rev Food Sci Nutr. 2022;62(7):1838-55.

128. Muir SW, Montero-Odasso M. Effect of Vitamin D Supplementation on Muscle Strength, Gait and Balance in Older Adults: A Systematic Review and Meta-Analysis. Journal of the American Geriatrics Society. 2011;59(12):2291-300.

129. Abshirini M, Mozaffari H, Kord-Varkaneh H, Omidian M, Kruger MC. The effects of vitamin D supplementation on muscle strength and mobility in postmenopausal women: a systematic review and meta-analysis of randomised controlled trials. J Hum Nutr Diet. 2020;33(2):207-21.

130. Antoniak AE, Greig CA. The effect of combined resistance exercise training and vitamin D(3) supplementation on musculoskeletal health and function in older adults: a systematic review and meta-analysis. BMJ Open. 2017;7(7):e014619.

131. Cheng SH, Chen KH, Chen C, Chu WC, Kang YN. The Optimal Strategy of Vitamin D for Sarcopenia: A Network Meta-Analysis of Randomized Controlled Trials. Nutrients. 2021;13(10).

132. Wei FL, Li T, Gao QY, Huang Y, Zhou CP, Wang W, Qian JX. Association Between Vitamin D Supplementation and Fall Prevention. Front Endocrinol (Lausanne). 2022;13:919839.

133. Prokopidis K, Giannos P, Katsikas Triantafyllidis K, Kechagias KS, Mesinovic J, Witard OC, Scott D. Effect of vitamin D monotherapy on indices of sarcopenia in community-dwelling older adults: a systematic review and meta-analysis. J Cachexia Sarcopenia Muscle. 2022;13(3):1642-52.

134. Habibi Ghahfarrokhi S, Mohammadian-Hafshejani A, Sherwin CMT, Heidari-Soureshjani S. Relationship between serum vitamin D and hip fracture in the elderly: a systematic review and meta-analysis. J Bone Miner Metab. 2022;40(4):541-53.

135. Ling Y, Xu F, Xia X, Dai D, Xiong A, Sun R, et al. Vitamin D supplementation reduces the risk of fall in the vitamin D deficient elderly: An updated meta-analysis. Clin Nutr. 2021;40(11):5531-7.

136. Cornish SM, Cordingley DM, Shaw KA, Forbes SC, Leonhardt T, Bristol A, et al. Effects of Omega-3 Supplementation Alone and Combined with Resistance Exercise on Skeletal Muscle in Older Adults: A Systematic Review and Meta-Analysis. Nutrients. 2022;14(11).

137. Rondanelli M, Perna S, Riva A, Petrangolini G, Di Paolo E, Gasparri C. Effects of n-3 EPA and DHA supplementation on fat free mass and physical performance in elderly. A systematic review and meta-analysis of randomized clinical trial. Mech Ageing Dev. 2021;196:111476.

138. Delpino FM, Figueiredo LM. Supplementation with omega-3 and lean body mass in the general population: A systematic review and meta-analysis. Clin Nutr ESPEN. 2021;44:105-13.

139. Camargo LDR, Doneda D, Oliveira VR. Whey protein ingestion in elderly diet and the association with physical, performance and clinical outcomes. Exp Gerontol. 2020;137:110936.

140. Hengeveld LM, de Goede J, Afman LA, Bakker SJL, Beulens JWJ, Blaak EE, et al. Health Effects of Increasing Protein Intake Above the Current Population Reference Intake in Older Adults: A Systematic Review of the Health Council of the Netherlands. Adv Nutr. 2022;13(4):1083-117.

141. Rus GE, Porter J, Brunton A, Crocker M, Kotsimbos Z, Percic J, et al. Nutrition interventions implemented in hospital to lower risk of sarcopenia in older adults: A systematic review of randomised controlled trials. Nutr Diet. 2020;77(1):90-102.

142. Bai GH, Tsai MC, Tsai HW, Chang CC, Hou WH. Effects of branched-chain amino acid-rich supplementation on EWGSOP2 criteria for sarcopenia in older adults: a systematic review and meta-analysis. Eur J Nutr. 2022;61(2):637-51.

143. Morgan PT, Harris DO, Marshall RN, Quinlan JI, Edwards SJ, Allen SL, Breen L. Protein Source and Quality for Skeletal Muscle Anabolism in Young and Older Adults: A Systematic Review and Meta-Analysis. J Nutr. 2021;151(7):1901-20.

144. Yang JM, Luo Y, Zhang JH, Liu QQ, Zhu Q, Ye H, et al. Effects of WB-EMS and protein supplementation on body composition, physical function, metabolism and inflammatory biomarkers in middle-aged and elderly patients with sarcopenic obesity: A meta-analysis of randomized controlled trials. Exp Gerontol. 2022;166:111886.

145. Coelho-Junior HJ, Calvani R, Picca A, Tosato M, Landi F, Marzetti E. Protein Intake and Frailty in Older Adults: A Systematic Review and Meta-Analysis of Observational Studies. Nutrients. 2022;14(13).

146. Martínez-Arnau FM, Fonfría-Vivas R, Cauli O. Beneficial Effects of Leucine Supplementation on Criteria for Sarcopenia: A Systematic Review. Nutrients. 2019;11(10).

147. Guo Y, Fu X, Hu Q, Chen L, Zuo H. The Effect of Leucine Supplementation on Sarcopenia-Related Measures in Older Adults: A Systematic Review and Meta-Analysis of 17 Randomized Controlled Trials. Front Nutr. 2022;9:929891.

148. Lee SY, Lee HJ, Lim JY. Effects of leucine-rich protein supplements in older adults with sarcopenia: A systematic review and meta-analysis of randomized controlled trials. Arch Gerontol Geriatr. 2022;102:104758.
